# The Role of Porphyrinoid Photosensitizers for Skin Wound Healing

**DOI:** 10.3390/ijms22084121

**Published:** 2021-04-16

**Authors:** Mariana C. S. Vallejo, Nuno M. M. Moura, Ana T. P. C. Gomes, Ana S. M. Joaquinito, Maria Amparo F. Faustino, Adelaide Almeida, Idalina Gonçalves, Vanda Vaz Serra, Maria Graça P. M. S. Neves

**Affiliations:** 1 LAQV-REQUIMTE, Department of Chemistry, University of Aveiro, 3810-193 Aveiro, Portugal; mariana.vallejo@ua.pt (M.C.S.V.); a.joaquinito@ua.pt (A.S.M.J.); 2 CESAM, Department of Biology, University of Aveiro, 3810-193 Aveiro, Portugal; ana.peixoto@ua.pt (A.T.P.C.G.); aalmeida@ua.pt (A.A.); 3 CICECO, Department of Materials and Ceramic Engineering, University of Aveiro, 3810-193 Aveiro, Portugal; idalina@ua.pt; 4 Centro de Química Estrutural, Instituto Superior Técnico, Universidade de Lisboa, Av. Rovisco Pais 1, 1049-001 Lisboa, Portugal; vanda.serra@tecnico.ulisboa.pt

**Keywords:** wound healing, photosensitizer, ALA, protoporphyrin-IX, porphyrins, chlorins, phthalocyanines, photodynamic inactivation, reactive oxygen species, skin, tissue regeneration

## Abstract

Microorganisms, usually bacteria and fungi, grow and spread in skin wounds, causing infections. These infections trigger the immune system and cause inflammation and tissue damage within the skin or wound, slowing down the healing process. The use of photodynamic therapy (PDT) to eradicate microorganisms has been regarded as a promising alternative to anti-infective therapies, such as those based on antibiotics, and more recently, is being considered for skin wound-healing, namely for infected wounds. Among the several molecules exploited as photosensitizers (PS), porphyrinoids exhibit suitable features for achieving those goals efficiently. The capability that these macrocycles display to generate reactive oxygen species (ROS) gives a significant contribution to the regenerative process. ROS are responsible for avoiding the development of infections by inactivating microorganisms such as bacteria but also by promoting cell proliferation through the activation of stem cells which regulates inflammatory factors and collagen remodeling. The PS can act solo or combined with several materials, such as polymers, hydrogels, nanotubes, or metal-organic frameworks (MOF), keeping both the microbial photoinactivation and healing/regenerative processes’ effectiveness. This review highlights the developments on the combination of PDT approach and skin wound healing using natural and synthetic porphyrinoids, such as porphyrins, chlorins and phthalocyanines, as PS, as well as the prodrug 5-aminolevulinic acid (5-ALA), the natural precursor of protoporphyrin-IX (PP-IX).

## 1. Introduction

**Figure 1:**
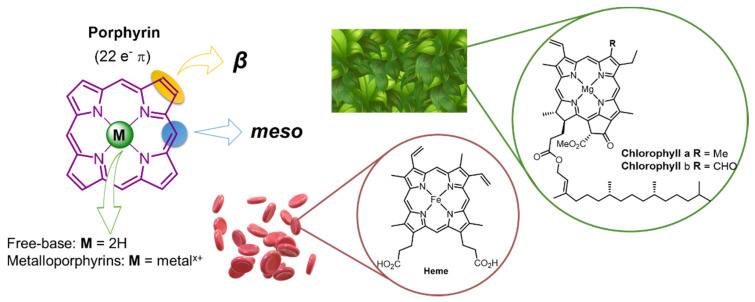
General structure of the porphyrinic nucleus and natural derivatives, such as chlorophyll *a* and *b*, and heme.

Porphyrins and analogs are a class of *N*-heterocyclic macrocycles well-known for their mediation in important biological functions, such as respiration and photosynthesis, but also by their role in a wide range of clinical and non-clinical applications that have high impacts on human life [1,2,3]. Natural tetrapyrrolic macrocycles like heme (iron(II) complex of PP-IX) and chlorophylls (Figure 1), but also the synthetic porphyrins and analogs ones (e.g., benzoporphyrins, chlorins, phthalocyanines) [4,5] are recognized to present unique structural, physicochemical, and photochemical features to be used in the development of dyes for dye-sensitized solar cells (DSSC) [6,7,8,9,10,11,12,13], (chemo) sensors [14,15,16,17,18,19,20], (photo)catalysts [21,22,23,24,25,26,27], biomarkers [14,28,29] or as therapeutic photosensitizers (PS) [30,31,32,33,34,35,36,37,38,39,40], among other applications [1].

**Figure 2:**
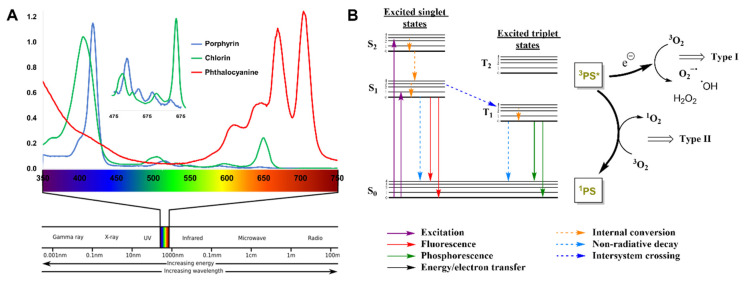
(**A**) Electromagnetic spectrum and typical absorption spectrum of porphyrins (blue line), chlorins (green line) and phthalocyanines (red line). (**B**) Simplified Jablonski diagram and reactive oxygen species (ROS) generation.

The strong absorption usually exhibited by these highly conjugated aromatic compounds in the visible region (Figure 2A) and their ability to generate reactive oxygen species (ROS), such as oxygen singlet (^1^O_2_), under light irradiation and in the presence of dioxygen (Figure 2B) are particularly relevant for their success to mediate the photodynamic process in photodynamic therapy (PDT) against tumoral cells [33,36,41,42], but also toward non-oncological pathologies, such as age-related macular degeneration. In the last two decades, porphyrinoids (either as free-base or coordinated with different metals) have been recognized as highly efficient PS to eradicate a broad range of microorganisms including drug-resistant strains using the same concept of PDT [31,37,43,44,45,46,47,48,49,50].

In this approach, usually referred to as photodynamic inactivation of microorganisms (PDI) or antimicrobial photodynamic therapy (aPDT), the PS, after being activated with light at an appropriate wavelength, is promoted to an excited singlet state (Figure 2). When returning to its stable ground state, the excess energy can be released by emitting light in the form of fluorescence or by heat production (internal conversion). Alternatively, the excited ^1^PS* can undergo the so-called “intersystem crossing (ISC) process”, affording the excited triplet state (^3^PS*). In this excited state, the PS can transfer their energy directly to dioxygen (^3^O_2_) (Type II mechanism), returning to the singlet ground state (^1^PS) and/or undergoes electron transfer reactions (Type I mechanism) to biological substrate molecules, leading to the formation of radicals and consequently, other reactive oxygen species (ROS), such as hydroxyl radical (OH), superoxide anion radical (O_2_^−^), and hydrogen peroxide (H_2_O_2_) (Figure 2B) [51,52,53].

**Figure 3:**
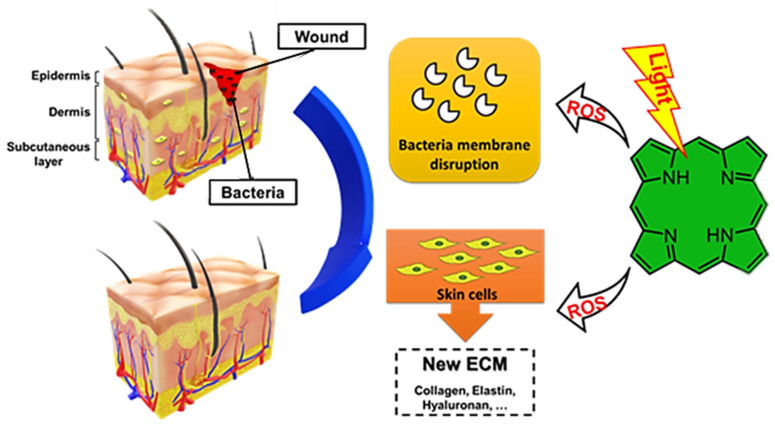
Schematic representation of tetrapyrrolic macrocycles photosensitizers (PS) contribution for wound healing.

Recently, the potential of PDT for tissue regeneration has caught the attention of many research groups concerning the role of non-porphyrinoid PS for skin wound healing, as was recently highlighted in our previous review [54]. In that review article, it is discussed that the PDT process not only enhances the mitochondrial activity, respiratory chain, and ATP synthesis [52,55], but also recruits important metalloproteinases (MMP)/growth factors (GF) and activate signal-regulated kinases [56,57] that are critical for the differentiation and proliferation of skin cells (such as fibroblasts) and extracellular matrix (ECM) components (such as elastin and collagen) [53,56,57,58] (Figure 3). However, bacterial infections should be considered since they are one of the major issues during skin wound healing and responsible for unsuccessful regenerative processes [59]. This is the main reason that makes PDI an attractive approach to both eradicate bacterial infections *in situ* and promote the local proliferation of healthy tissue. One of the major challenges is fine-tuning the structural and photophysical properties of PS to have strong absorption in the ideal “phototherapeutic window” (wavelengths ranging from 600 to 800 nm) where the light penetration in the tissue is high. In fact, most of the porphyrinoid-based PS described as effective for wound healing absorb in this region (Figure 2).

The PS studies comprised (i) first-generation, consisting of mixtures obtained from natural occurring porphyrins (e.g., hematoporphyrin derivative, Photofrin); (ii) second-generation, such as porphyrins, chlorins, benzoporphyrins and phthalocyanines with pure structures; and (iii) third-generation, resulting from the combination of second-generation PS with specific delivery carriers, such as antibodies, liposomes, polymers, nanoparticles, and micelles to overcome the drawbacks of the other two PS generations, such as low accumulation on the desired site, self-aggregation and low solubility in aqueous media [46,59,60]. 

The increasing number of publications embracing tetrapyrrolic macrocycles as PS for skin wound healing prompts us to compile these works separate from our previous publication where the non-porphyrinoids PS were the target [54]. Herein, this review will focus the use of tetrapyrrolic macrocycles, namely porphyrins, chlorins, and phthalocyanines, as PS for skin wound healing, with some of them clinically approved (e.g., Hematoporphyrin derivative, Photofrin^®^, Foscan^®^, Fotoditazin^®^, Visudyne^®^) but also on the approved prodrugs 5-aminolevulinic acid (Levulan^®^) and derivatives (Metvix^®^ (methyl ester derivative) and Hexvix^®^ (hexyl ester derivative)) as precursors of endogenous PP-IX. This review is organized according to the structural features of each PS and, to facilitate the discussion, the macrocycles obtained from natural sources were separated from the synthetic ones.

## 2. 5-Aminolevulinic Acid and Derivatives as Precursors of Endogenous Protoporphyrin-IX

**Figure 4:**
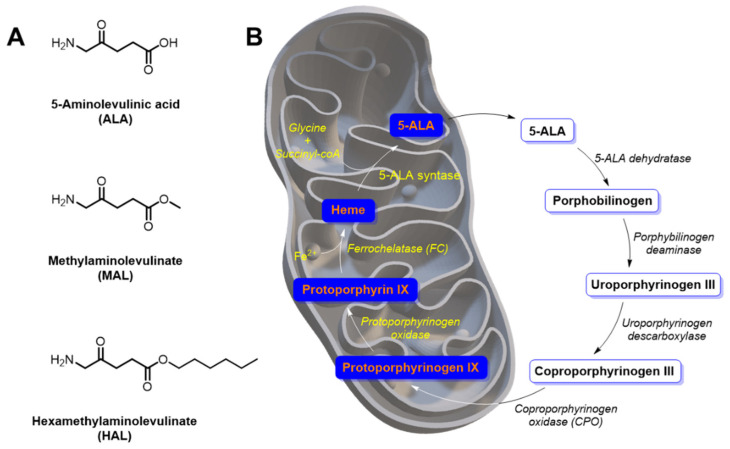
(**A**) Structure of the prodrugs 5-ALA and its derivatives MAL and HAL; (**B**) schematic representation of the heme cycle.

Under the context that PDT can be beneficial in wound healing, special attention is being given to the prodrug 5-aminolevulinic acid (5-ALA or just ALA) and to its ALA alkyl esters (e.g., ALA methyl ester) due to their recognized efficacy to treat different oncological (e.g., squamous cell tumors and actinic keratosis) and non-oncological skin malignancies (e.g., acne, psoriasis, cutaneous leishmaniasis) [61,62,63,64,65,66]. The 5-ALA is a key intermediate in the enzymatic controlled metabolic pathway leading to PP-IX and then to heme (Figure 4); if an excess of ALA is provided to the cells, there will be an extra accumulation of PP-IX in the cell and, consequently, under irradiation, this porphyrin can act as the truthful PS (Figure 4) [67,68,69]. When ALA alkyl esters are used, deeper penetration in the tissues is observed, caused by their high lipophilicity, allowing esterases to act at the target tissue and to perform the dealkylation step.

In 2001, the group of Gupta selected the prodrug 5-ALA to evaluate if the combined action of low-energy lasers (recognized to have a wound healing positive effect [70,71]) and endogenous PS improved the healing of excisions created in rats [72]. In this study, the irradiations were performed using the laser He-Ne (3 J/cm^2^) alone or in combination with the laser Nd:YAG (30 J/cm^2^), using the treatment with a hematoporphyrin derivative (HpD, *vide infra* Figure 15) as control (Table 1). For comparison, the wounds treated with laser He-Ne (3 J/cm^2^) in the presence of 5-ALA (Test-1) and HpD (Test-3) were compared with not treated wounds (control-1); wounds treated with ALA (control-2); or wounds treated with HpD (control-3) in the absence of light (dark controls). The animals from Test-2 and Test-4 received an extra light dose of 30 J/cm^2^ simultaneously with the 3 J/cm^2^.

The results from Table 1 concerning wound closure pattern show that the best performance was attained from the animals treated with ALA and irradiated with He-Ne laser (3 J/cm^2^) with a complete closure of the wound on the 13 ± 1 day (entry 4), followed closely by the group treated with HpD and exposed to the combined action of He-Ne (3 J/cm^2^) and Nd-YAG lasers (30 J/cm^2^) (entry 7). The authors assumed that further studies are required to explain the reasons for the results achieved with ALA and the combined action of both laser treatments (entry 5) which slowed down the healing process from 13 to 17 days. It was also found that the wounds treated with administered ALA revealed a higher epidermal closure and dermal healing when compared with other groups. Although this study suggested that the photodynamic action quickens the wound healing process on rats, the lack of controls treated only with light does not allow for evaluating the real photodynamic effect. 

**Figure 5:**
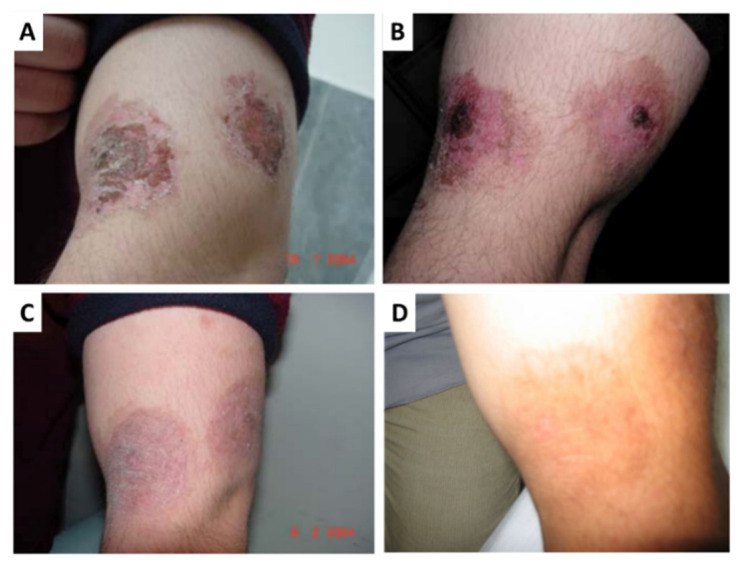
Healing evolution of an ulcerated wound after PDI in one patient with leishmaniasis: (**A**) 1st session, (**B**) 2nd session, (**C**) 4th session, and (**D**) four months after the PDI (figure adapted from [75] with permission of World Health Organization).

The conventional treatments of cutaneous Leishmaniasis (caused by the flagellated protozoa, *Leishmania major* and *Leishmania tropica*) in humans are usually associated with a high risk of recurrence, poor cosmetic outcomes, development of resistance and symptoms related to different diseases, such as malaise and anorexia, among others [73]. So, considering that porphyrins in combination with menadione (vitamin k) induced in vitro selective destruction of amastigotes in the macrophages [74], Ghaffarifar and his group evaluated the effect of PDI mediated by ALA to treat five patients with confirmed cutaneous Leishmaniasis lesions [75]. The lesions under occlusion were treated with an ALA formulation (10% ALA in a water-in-oil emulsion) and after 4 h, were irradiated with a red light (570–670 nm) at an irradiance of 150 mW/cm^2^, delivering in each treatment session of 21 min a total light dose of 189 J/cm^2^. This treatment was repeated once a week for a month (4×) and the selective accumulation of ALA in the lesions was confirmed by the fluorescence emitted by the ALA-induced porphyrins after exposure to light. The results revealed amastigotes eradication after one treatment session in the lesions of three patients and after two sessions in the lesions of the remaining two patients. It was also highlighted that during the following-up four months, there was no recurrence of amastigotes, and after one month of treatment, the healing and cosmetic outcomes among all the patients were considered excellent (Figure 5). Although the authors recognized that the number of patients was limited, and no comparison with conventional treatment was performed, the approach should be considered an attractive antiparasitic therapeutic option since the adjacent normal tissues were not affected and, in contrast to systemic treatments, the risk of toxicity is minimal.

**Figure 6:**
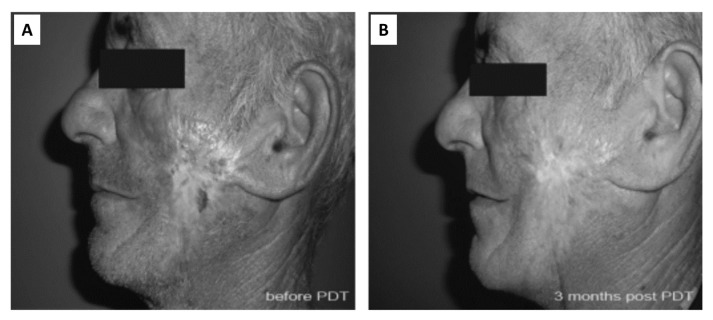
(**A**) Ulcerated lesion of cutaneous leishmaniasis on the left cheek before treatment, and (**B**) three months post-treatment (figure adapted from [76] with permission from John Wiley and Sons).

In 2011, another promising achievement related to the use of PDI mediated by 5-ALA to treat cutaneous leishmaniasis was reported by Evangelou and coworkers [76]. The authors used the approach to treat a patient with long-standing cutaneous leishmaniasis with an extensive manifestation on the left cheek where surgery was unsuitable due to the extent of the disease (Figure 6). The lesion was injected with 5-ALA and after 4 h, was irradiated with red light at 630 nm at an irradiance of 45 mW/cm^2^ (total light dose of 100 J/cm^2^). This procedure was repeated three times at weekly intervals, and it was accompanied by a prompt healing response (Figure 6) and absence of any recurrence during the follow-up (after two years, the patient remained clinically clear of disease). This achievement seems to indicate that intralesional administration of ALA-PDI can be an alternative to treat recurrent cutaneous leishmaniasis, equally or even more effective than superficial application due to the enhanced light tissue penetration. Nevertheless, the true efficacy of this treatment modality can only be established in appropriately designed studies with larger patient groups.

Nowadays, ulcers are one of the most challenging types of wounds, due to their association with persistent localized infections bringing a long-lasting process of healing and high monetary costs to the patient. Chronic ulcers affect around 1% of the adult population, thus being one of the focuses of the skin regeneration research field [77].

In 2007, Clayton reported that PDI mediated by 5-ALA also merits a place in treating leg-infected ulcers. The group considered the results obtained with a patient with a chronic recalcitrant venous ulceration (with eventual methicillin-resistant *Staphylococcus aureus* (MRSA) colonization) on her lower leg, where various conventional treatments (e.g., topical antiseptics such as potassium permanganate, silver nitrate and different bacteriostatic dressings) had proved unsuccessful [78]. The PS was topically applied to the lesion and then the ulcer was irradiated with red light at 633 nm twice weekly over four weeks. It was observed that the treatment was well tolerated with minimal discomfort to the patient, even without the use of topical local anesthesia. The authors referred that the significant improvement observed in the ulcer was correlated with the negative skin swabs obtained from the lesion. Additionally, the authors suggested that PDI may also reduce requirements for systemic antibiotics in the management of skin infections, and it was recognized that further studies into PDI and its antimicrobial potential are needed.

**Figure 7:**
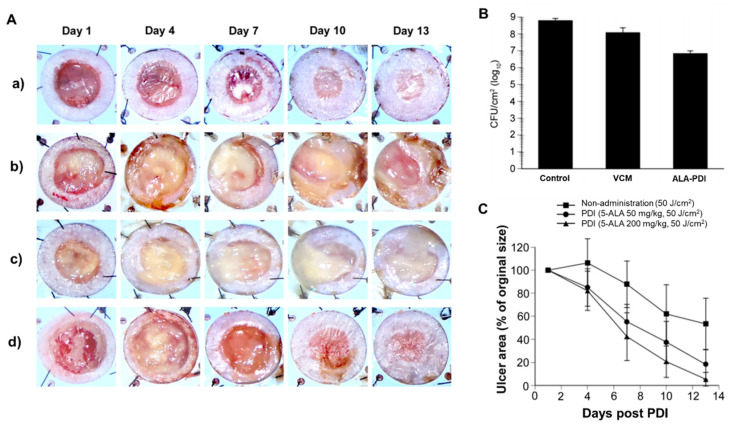
(**A**) General morphologies of cutaneous ulcers were imaged on days 1, 4, 7, 10, and 13 in (**a**) mice without MRSA, (**b**) mice with MRSA, (**c**) vancomycin (VCM)-treated mice, and (**d**) PDI-treated mice (200 mg/kg 5-ALA and a total light dose of 50 J/cm^2^). (**B**) MRSA counts (in CFU/mL) before and after PDI and VCM treatment. (**C**) Evolution of ulcer area of treated with blue light (410 nm) with a total light dose of 50 J/cm^2^, treated with 0, 50 and 200 mg/kg of 5-ALA (figure adapted from [79] under Creative Commons attribution License).

The possibility of using a 410 nm wavelength light-emitting diode (Blue LED) and 5-ALA to treat MRSA infected cutaneous ulcers induced in the backs of rats was considered by Tsuruta in 2014 [79]. In this study, after confirming that ALA-PDI with a blue LED at an irradiance of 164.5 mW/cm^2^ exerts an in vitro antibacterial effect on MRSA, the infected wounds were injected intraperitoneally with different concentrations of 5-ALA (0, 50, or 200 mg/kg) and irradiated with the blue LED (total light dose of 50 J/cm^2^) (Figure 7A–C). For comparison, three other treatment groups were performed: non-infected wounds, non-treated infected wounds, and wounds treated with vancomycin (VCM) (Figure 7B).

The results showed that the best achievement was reached when the daily treatment was performed in the presence of 200 mg/kg of 5-ALA under blue LED irradiation. It was observed that at day 13, the mean ulcer area of the PDI-treated mice was like that observed for ulcers without MRSA infection, demonstrating that epithelialization was accelerated after PDI, but not with the administration of VCM. 

The results corroborate that ALA-PDI, besides the direct antimicrobial effect, has also some influence on the immune system involved in the acceleration of wound healing.

**Figure 8:**
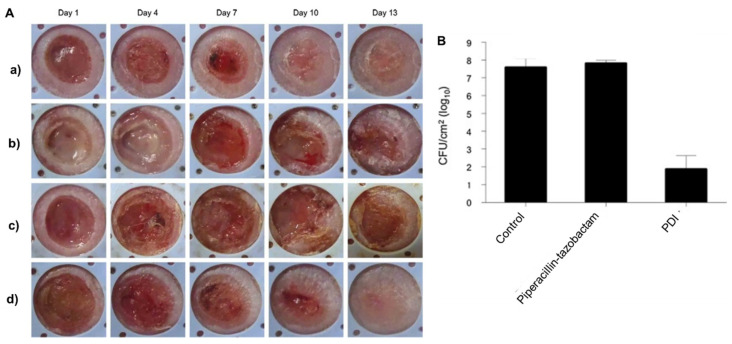
(**A**) Wounds were photographed on days 1, 4, 7, 10, and 13 in mice without *P. aeruginosa* infection (**a**), mice with *P. aeruginosa* infection (**b**), mice with *P. aeruginosa* infection treated with piperacillin-tazobactam (**c**), and mice with *P. aeruginosa* infection treated with PDI (**d**). (**B**) Bacterial count in ulcer tissue before and after PDI or treatment with piperacillin-tazobactam, using not treated wounds as the control (figure adapted from [80] with permission from Elsevier).

Later, the group extended the approach to treat cutaneous wounds infected with the Gram-negative bacterium *Pseudomonas aeruginosa* using disodium ethylenediamine tetraacetic acid (EDTA) [80]. The study showed that the presence of the EDTA salt was crucial for an efficient accumulation of 5-ALA-derived PP-IX in the bacteria and, consequently, for the required bactericidal effect. The EDTA/5-ALA (0.5% 5-ALA, 0.005% EDTA) formulation was applied topically in the wounds and treated using blue LED light at 410 nm at an irradiance of 164.5 mW/cm^2^ and a total light dose of 9 J/cm^2^. The results were compared with the ones obtained from non-infected wounds, wounds infected with *P. aeruginosa* but not treated, and infected wounds treated with the piperacillin-tazobactam antibiotic (Figure 8). The monitorization for 13 days allowed to conclude that the photodynamic treatment using 5-ALA combined with EDTA improved the healing in the infected wounds. Once more, these results corroborated that besides sterilization, PDI has some role in the acceleration of the healing of the infected cutaneous wounds.

**Figure 9:**
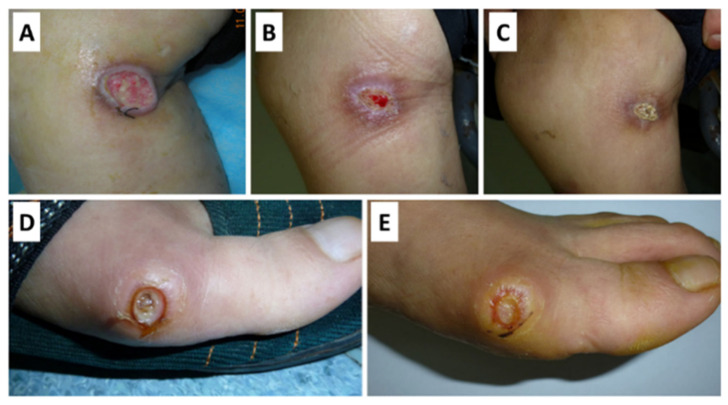
Patient 1: skin ulcer in the right leg with yellow secretions before treatment had healed three days after the second PDI treatment (**A**–**C**). Patient 2: skin ulcer surface was significantly cleaner, and the size was reduced three days after the second PDI treatment (**D**,**E**) (figure adapted from [69] with permission from Springer Nature).

In 2015, Lei and his coworkers reported the promising results obtained from a clinical study where PDI mediated by 5-ALA was used to treat patients with *P. aeruginosa*-infected chronic skin ulcers in lower limbs (Figure 9) [69]. In this study, one group of 13 patients was enrolled for the 5-ALA-mediated PDI (20% 5-ALA solution), using red light (630 nm, total light dose of 80 J/cm^2^), while another group, with the same number of patients, was treated only with red light. In both cases, the treatment was performed once a week for two weeks. At the seventh day post-treatment, the mean ulcer area in the red-light group was reduced from 11.85 ± 6.83 to 7.8 ± 4.9 cm^2^ (ca. 42% of affected area reduction), and that of the PDI group (5-ALA and red light) from 12.72 ± 8.58 to 3.4 ± 3.4 cm^2^ (ca. 73% of affected area reduction). These results are in accordance with the significant reduction in the bacteria levels on the surfaces of the ulcers in the PDI group after 24 h post-treatment, when compared with the bacteria level of the ulcers just treated with red light. Although the results obtained were promising as it is patent by the two examples shown in Figure 9, it was recognized that the approach requires further validation using a large sample size in clinical trials, and the specific mechanism behind the treatment merits further studies. 

In the same period, Cappugi and his group selected a galenic gel-based formulation containing 16% of 5-ALA to treat infected chronic venous ulcers (CVU) (with *Enterococcus faecalis* and/or *Staphylococcus aureus*) under PDI conditions of 19 pre-selected patients [81]. The formulation was topically applied onto the wound surface and after 3 h, the lesions were irradiated for 12 min with a red light at 618 nm (total light dose of 100 J/cm^2^). The same procedure was repeated once a week up to 10 times. Dressings with hyaluronic acid were applied and no other treatment was carried out during the study period. The results showed that the ulcers healed in 15 cases (78.9%) after an average of 6.8 PDI sessions (range 6.0–8.0). In the other four cases, the best achievement was observed after 10 PDI sessions and it was suggested that these patients undergo an autologous skin graft. Nonetheless, no pain or side effects were recorded in all patients and the results seem to corroborate that PDI with 5-ALA and red light represents a good therapeutic alternative to treat refractory CVU.

**Figure 10:**
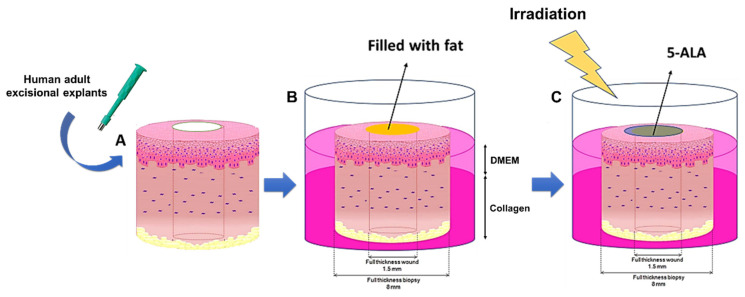
Schematic representation of a full-thickness wound healing organ culture (WHOC) model of 1.5 mm. The wound was created from an explant of human skin using a biopsy punch (**A**), which is then filled with fat to create a scaffold (**B**). The surface of the wound was then covered with the photosensitizers (PS) to initiate the photodynamic treatment (**C**) (figure adapted from [68] with permission from John Wiley and Sons).

Garcia and coworkers selected also PDI mediated by 5-ALA to evaluate the potentiality of an optimized wound healing organ culture (WHOC) to be used as a model for investigating the process of cutaneous repair, as an alternative to the use of patients or animals [68]. In the construction of these ex vivo models, the authors used skin explants removed from healthy patients with partial or full-thickness wounds obtained by using tools such as a scalpel of a biopsy punch. In the photodynamic studies, the full-thickness excisional wounds (doughnut-shaped model) were filled with either subcutaneous fat (excised from the harvested tissue) or the BD Matrigel^TM^ scaffold, and the irradiations in the presence of 5-ALA were performed with red light-emitting diodes (LEDs) at 633 nm (total light dose of 20 J/cm^2^) (Figure 10). The procedure was repeated once more after 3 days, and the wound model evolution was monitored for 14 days. The results, when compared with the untreated WHOCs, revealed that photodynamic treatment increases re-epithelialization, ECM reconstruction and remodeling. So, the optimized WHOC model can offer an efficient ex vivo alternative to evaluate the efficiency of other photodynamic candidate therapeutic agents in skin wound healing.

The high specificity of the methyl ester of 5-ALA (methyl aminolevulinate; MAL) for cancerous cells and its approval to treat actinic keratosis prompted Berking et al. to select this ester for the photodynamic treatment of 15 patients with actinic cheilitis [82]. This pathology is a subtype of actinic keratosis resulting from long-term exposures to sun and the lesions are, in general, localized in the lower lips. Moreover, the group considered that the previously reported achievements using the photodynamic effect mediated by ALA or MAL involved a limited number of patients or a high number but without histopathologic evaluation of the lesion [83,84,85]. For this treatment, MAL was administered topically into the lesions and irradiated with red light (630 nm) at an irradiance of 68 mW/cm^2^ for 12 min (total light dose of 49 J/cm^2^) at intervals of one week. The wounds were monitored for 14 days, and the results revealed that in all patients, superficial desquamation and hemorrhagic crusting were developed after two to four days of treatment. Most of the patients experienced a marked improvement of their condition with a complete (47%) or a partial cure (47%) after two sessions of PDT; however, in the follow-up after three months, the histopathologic analysis revealed complete healing in 38% of patients whereas in 62% patients histomorphologic features of actinic cheilitis were still detected.

The authors concluded that photodynamic action mediated by MAL after an optimization of the treatment protocol to achieve more acceptable response rates can be a promising approach to treat patients with actinic cheilitis, namely those with higher risks for invasive therapeutic strategies. 

The possibility of using MAL as a precursor of PP-IX was also considered by the Devirgiliis group to treat a patient with chronic venous ulceration infected by the two Gram-positive bacteria *S. aureus* and *E. faecalis* and not responsive to conventional treatments [86]. The lesion was treated with a cream containing MAL and after 3 h was irradiated with a LED light at 630 nm (total light dose of 37 J/cm^2^). This treatment was repeated once a week for a total of four treatments (one month). After this period, the cutaneous swabs became negative, and it was observed that there was a significant clinical improvement. So, the infection by Gram-positive bacteria was eliminated by MAL mediated PDI and the approach seemed to be responsible by promoting the wound healing by enhancing re-epithelialization, granulation tissue formation, and collagen organization. Therefore, although further studies would be necessary, it can be assumed that the PDI approach represents a valid option to treat infected chronic ulcers. 

In 2014, Mills et al. reported the first study concerning humans where the impact of the photodynamic action induced by MAL on clinical and microscopic parameters of cutaneous excisional wounds was evaluated [67]. In the study, besides the cosmetic appearance, the authors gave a particular focus on the production of transforming growth factor (TGF) isoforms TGF-β1, TGF-β2 and TGF-β3, and metalloproteinases (MMP) 1, 2, and 9 due to their roles at various stages of the healing process [54,87,88].

**Figure 11:**
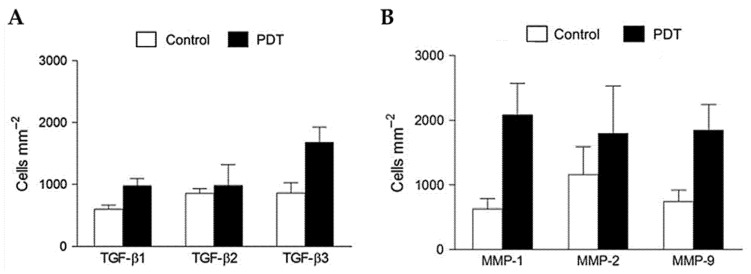
(**A**) Quantification showed significantly higher numbers of transforming growth factors TGF-β1- and TGF-β3-producing cells in treated wounds compared with controls three weeks post wounding (*n* = 6–8). (**B**) Quantification showed significantly higher numbers of metalloproteinases MMP-1- and MMP-9-producing cells in treated wounds compared with controls three weeks post wounding (*n* = 6, except for MMP-2, *n* = 4) (figure from reference [67] with permission of John Wiley and Sons).

In the PDT assays, the wounds were topically treated with MAL and then irradiated with red light at 630 nm at an irradiance of 80 mW/cm^2^ for 9 min with a total light dose of 43 J/cm^2^. The results showed that after three weeks, the treated wounds, when compared with the non-treated ones, were smaller, and the number of transforming growth factor TGF-β3-producing cells (responsible by the reduction of scarring through the promotion of collagen organization) was significantly higher in the PDI groups with an important TGF–β3:β1 ratio (Figure 11A). In the MAL-PDT-treated wounds, there was a significant increase of the metalloproteinases MMP-1 and MMP-9 with an important role in the matrix remodeling (Figure 11B). Although the levels of TGF-β2 (responsible for promoting inflammatory cell recruitment and collagen production like TGF-β1), remained statistically unchanged in both groups, the results indicate a positive stimulation of the inflammatory and remodeling phases of wound healing. It was recognized that optimized protocols must be extended to the analogous clinical situation of surgical excision.

**Figure 12:**
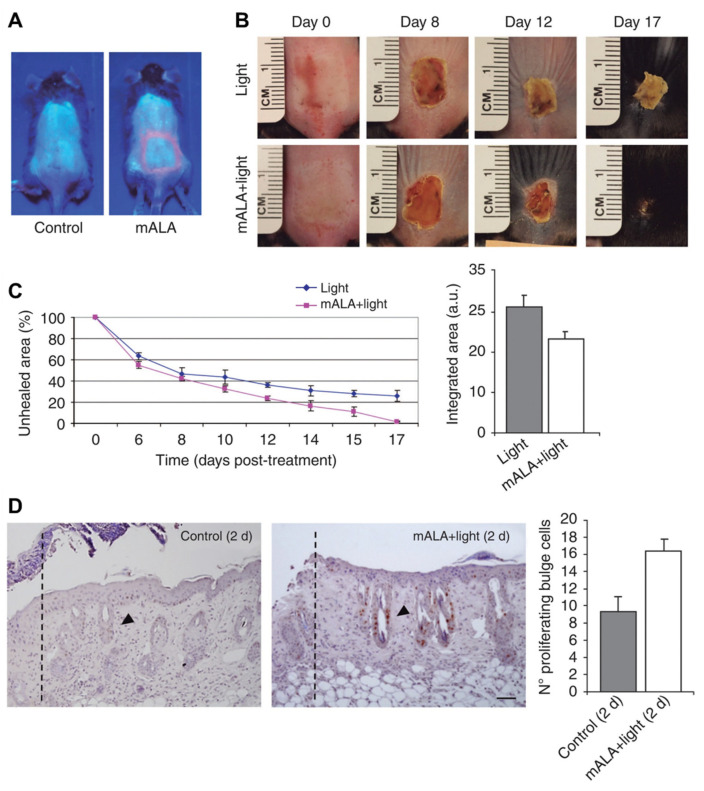
(**A**) Protoporphyrin-IX production induced by MAL (=“mALA”) in burn-injured regions in treated animals as compared with control samples.(**B**) Burn healing evolution in the animals’ skin. (**C**) Time-course quantification of burned areas showing accelerated burn healing in MAL (“mALA”) + light-treated animals comparatively to control group. (**D**) Histological images showing stained (blue) proliferative cells (arrowheads), and hair follicles located in the adjacent area of the burn boundary (dotted lines) in MAL (“mALA”) + light as compared with control samples. Right panel: quantification of the number of proliferating cells in the three hair follicles closest to the burn boundary (figure adapted from [89] with permission from Elsevier).

The combination of MAL (in this study referred as “mALA”) and red light (636 nm) was also used to study the impact of transient ROS production to promote cell proliferation in mouse skin and in the hair follicle stem cell niche to improve hair growth and wound healing [89]. The photodynamic treatments were conducted 2.5 h after topically applied MAL (“mALA”) on 2nd degree burns induced on mice skin. After red light irradiation (636 nm; total light dose of 2.5–10 J cm^−2^), it was observed that MAL (“mALA”) and red light significantly accelerated the healing process (Figure 12A–C); this regenerative effect was associated with extensive cell proliferation in the bulge region of the air follicle adjacent to the burned area (Figure 12D), indicating the activation of this stem cell niche. These results suggest that the activation of skin proliferation and the hair follicle stem cell niche by a transient ROS production is a molecular signal able to stimulate different homeostatic programs in the skin, including hair growth and tissue regeneration.

**Figure 13:**
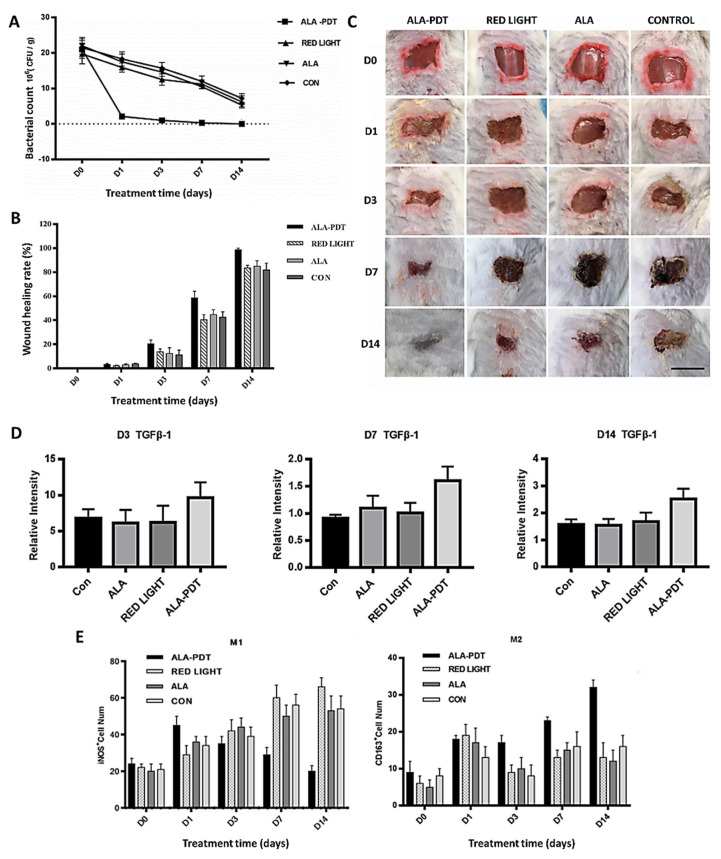
(**A**) Comparison of *P. aeruginosa* counting on wounds treated with 5-ALA and red light (ALA-PDT) and respective controls; (**B**) healing rate counted before treatment (D0) and on the 1st (D1), 3rd (D3), 7th (D7), and 14th (D14) day after different treatments; (**C**) wound pictures of the healing process (Scale bar indicate 1 cm); (**D**) western blot analysis of TGF-1 and (**E**) immunofluorescence staining analysis of M1 and M2 macrophages (figure adapted from [90] under Creative Commons Attribution License).

In 2020, the group of Lei studied the therapeutic effects of 5-ALA mediated PDI in mouse skin wounds infected with Gram-negative bacterium *P. aeruginosa* and its role at various stages of the healing process [90]. The assays involved four experimental groups: wounds not treated (control), wounds treated only with red light (630 nm) at an irradiance of 90 mW/cm^2^ for 10 min (total light dose of 54 J/cm^2^), wounds treated with 5-ALA (1.408 mol/L) in the absence of light, and wounds treated with ALA under red light irradiation (ALA-PDT). The wounds evolution was monitored for 14 days, and the results (Figure 13) revealed that ALA-PDT treatment significantly reduced the load of *P. aeruginosa* in the wounds (Figure 13A) and improved their healing rate when compared with the controls (Figure 13B,C). A gradual increase in the expression of growth factors like TGF-β-1 was also observed in wounds subjected to photodynamic action, comparatively to the remaining testing groups (Figure 13D). The results also showed that the photodynamic treatment (ALA-PDT) affected the polarization state of macrophages, activating and promoting macrophages from a M1 to a M2 phenotype (Figure 13E). The number of M1 macrophages on the 1st day was significantly increased in the ALA-PDT group being significantly lower on the 7th and 14th day, when compared with the controls, while the number of M2 macrophages in the ALA-PDT group on the 3rd, 7th, and 14th day, was significantly higher than that in the control group. This study is another important contribution showing the role of ALA in the photodynamic treatment to promote the healing in skin infected wounds by regulating inflammatory factors, collagen remodeling, and macrophages, besides, of course, its photokilling action towards bacteria.

## 3. Tetrapyrrolic Macrocycles with Natural Origin

**Figure 14:**
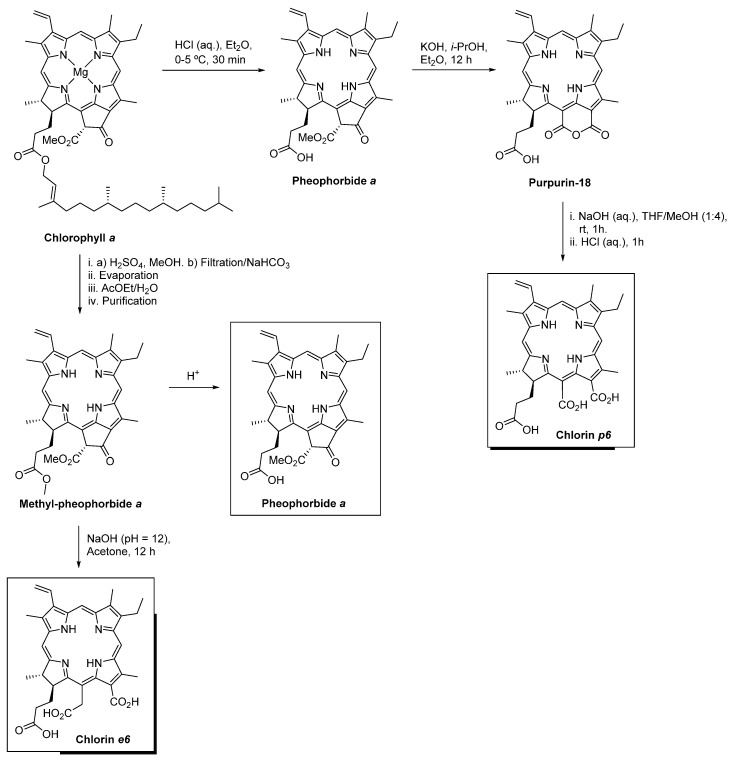
Synthetic routes used to prepare natural chlorin derivatives pheophorbide *a*, chlorin *e6*, and chlorin *p6* from chlorophyll *a*.

**Figure 15:**
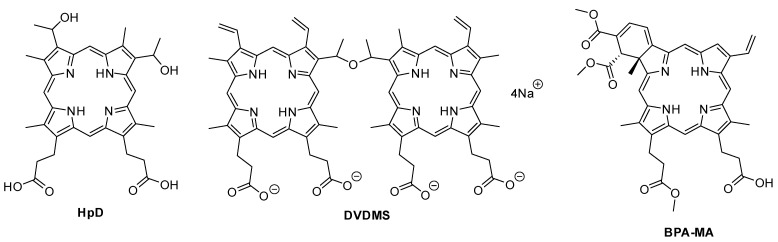
Structure of natural porphyrin derivatives hematoporphyrin (HpD), sinoporphyrin sodium salt (DVDMS), and benzoporphyrin mono-carboxylic acid (BPD-MA).

Most of the PS of natural origin selected by the researcher groups to evaluate the impact of PDI skin wound healing are based on chlorophyll *a* derivatives (Figure 14) and PP-IX and derivatives (Figure 15), due to their recognized efficacy under different clinical contexts [91].

Summarized in Figure 14 are the well-established synthetic pathways leading to some of those key chlorophyll *a* derivatives, such as pheophorbide *a*, chlorin *e6,* and chlorin *p6,* [5,92,93,94], which in some cases were used as a functional handle to obtain further PS with improved biological features. The formation of pheophorbide *a* can also be promoted by the action of chlorophyllase and Mg-dechelatase, leading first to chlorophyllide *a* and then to the free-base [95,96].

### 3.1. Pheophorbide a

**Figure 16:**
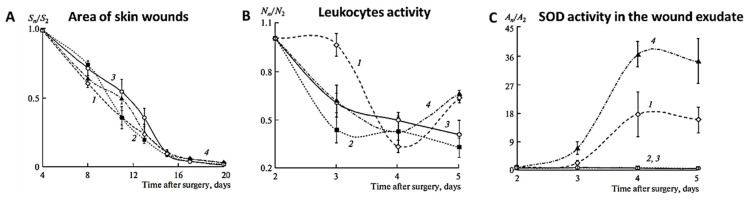
(**A**) Changes in wound area; (**B**) change in the relative number of leukocytes (N_n_/N_2_); and (**C**) in SOD activity (A_n_/A_2_) in the wound exudate from the second to the 15th day after making the wound; “n” in “X_n_” (X = S, N, A) is the correspondent activity on day 3–5, and “2” in “X_2_” (X = S, N, A) on day 2 from the start of the experiment; -lines: 1 = first group (control); 2 = second group (pheophorbide *a* + red laser irradiation), 3 = third group (protoporphyrin-IX + laser irradiation), 4 = fourth group (red laser irradiation) (figure adapted from [97] with permission from Springer Nature).

In 2010, Lyapina et al. compared the healing action of pheophorbide *a* and PP-IX towards aseptic incised skin wounds in rats under low-level laser irradiation (LLLT) [97]. The wounds treated with pheophorbide *a* (Group 2) or PP-IX (Group 3) were irradiated with a red laser light at 632.8 nm at an irradiance of 2.87 mW/cm^2^ and a total light dose of 1.72 J/cm^2^. The results were compared with non-treated wounds (Group 1) and just treated with light (Group 4) (Figure 16). The authors had taken into account the treatment effect in the wound sizes (Figure 16A), but also on leukocytes activity due to their role at the different stages of wound healing (inflammation, proliferation, and maturation of the granulation tissue) and superoxide dismutase (SOD) activity (Figure 16C,D). After five days, the wounds irradiated in the presence of both PS showed a decline in the amount and activity of leukocytes and on SOD activity, when compared with the controls. These results suggest that, in these groups, the wound healing inflammatory stage was substantially accelerated; the ability of PS to generate ROS upon irradiation can lead to an acceleration of wound cleansing. No significant differences were found between the effect of the two different PS (porphyrin versus chlorin) and, although on the 20th day all the groups achieved complete healing, the test groups (pheophorbide *a* or exogenous PP-IX + irradiation) showed a less pronounced cicatrization (smooth scars) than the control groups.

### 3.2. Chlorin e6

**Figure 17:**
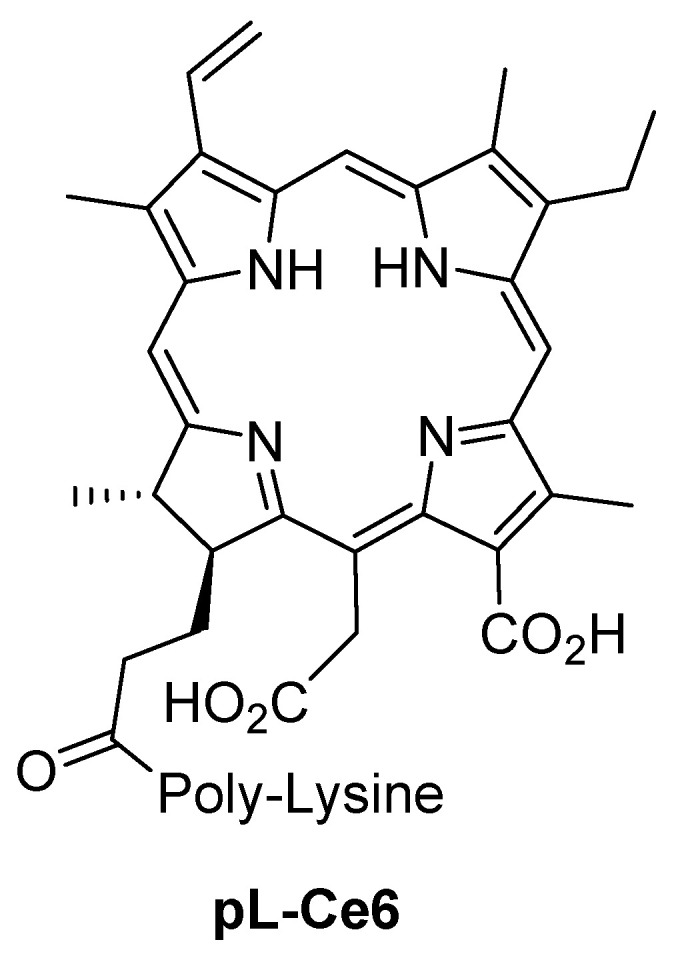
Structure of pL-C*e6* conjugate.

Hamblin and his co-workers developed a remarkable work for almost twenty years concerning the potential of chlorin *e6* (C*e6*) as PS under different contexts, namely for wound healing [98,99,100,101]. In fact, the aPDT healing ability has been a topic intensively revised in recent years by the group [102,103,104,105]. In some of these studies, the authors evaluated the efficiency of poly-L-lysine chlorin *e6* (pLC*e6*) conjugate (Figure 17) with results particularly relevant in the aPDT context [98,99,106]. In the report of 2002, a bioluminescent *Escherichia coli* strain (*E. coli* DH5α) was inoculated into excisional wounds on mice and treated with pL-C*e6* using a 665 nm laser light (1 W diode; at an irradiance of 100 mW/cm^2^) [98]. The viability of the *E. coli* was monitored by an optical technique using an intensified charge-coupled device (ICCD) camera, that allowed the monitoring of the bacterial viability in real-time in living animals. The results showed the loss of the luminescence signal only in the wounds treated with pL-C*e6* and light, indicating that *E. coli* was efficiently photoinactivated. Moreover, it was also found that PDI treated wounds healed as well as control wounds, showing that the photodynamic treatment did not damage the host tissue. This study also demonstrated the strength of an *in vivo* assay for infection in living animals [98].

**Figure 18:**
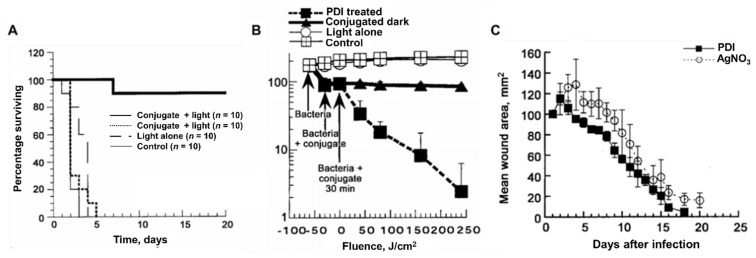
(**A**) Percentage of mice survival for different treatment procedures.; (**B**) luminescence signals from the infected wounds, before and after PDI treatment; and (**C**) time evolution of the mean areas of mice’s wounds treated with PDI or topical AgNO_3_ (figure adapted from [99] with permission from Oxford University Press).

The PDI efficacy was also evaluated using excisional mouse wounds infected with bioluminescent *P. aeruginosa* using the same pL-C*e6* conjugate as PS and under the same light irradiation procedure [99]. In this case, the bioluminescence was monitored by ICCD camera. The results showed that only the wounds treated with pL-C*e6* and light suffered a rapid loss of luminescence, which was dependent on the light dose (Figure 18B). This study also showed that only the mice group whose wounds were treated with pL-C*e6* and light had survived (Figure 18A). Moreover, the authors compared the effect of the antibacterial agent AgNO_3_ in the bacterial infection and wound healing with those achieved with PDI and found that PDI-treated wounds healed significantly faster than the AgNO_3_-treated wounds (Figure 18C). 

**Figure 19:**
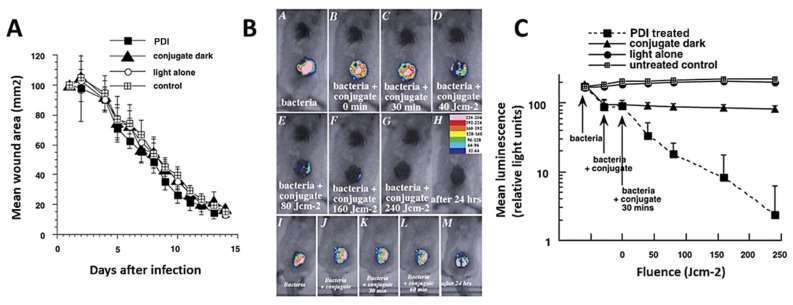
(**A**) Mean areas of wounds measured daily; (**B**) successive overlaid luminescence false-color images and monochrome LED images of mice bearing an excisional wound infected with 5 × 10^6^ luminescent *P. aeruginosa.* (Panels A–H). A representative mouse treated with pL-C*e6* conjugate and increasing light doses. (Panels I–M); and (**C**) mean pixel values of bioluminescence signals from infected wounds (figure adapted from [106] with permission from Elsevier).

Soon after, Hamblin’s group reported the PDI efficiency of pL-C*e6* to treat localized infections in wounds and soft tissue abscesses present in mice [106]. In this work, the authors selected two Gram-negative bioluminescence strains, *E. coli* and *P. aeruginosa*, and the Gram-positive bioluminescent *S. aureus*. For all bacteria, a light-dose dependent loss of luminescence was observed when pL-C*e6* was introduced into the infected tissue followed by illumination with red light, showing the efficient bacteria photoinactivation. This work also showed that the healing of PDI treated wounds was not inhibited by the PDI protocol (Figure 19A). The results achieved with bioluminescent *P. aeruginosa* are noteworthy. Not only were the bacterial loads in the wound sites significantly reduced, but also only the mice group subjected to the PDI protocol were able to survive from the infection by this highly fatal bacterium (Figure 19B,C). In what concerned the results achieved for the treatment of soft-tissue infections with *S. aureus* in mice, despite the treated infected legs had been reduced by >99% (reduction of 2 log) compared to the untreated contralateral legs, in most of the cases, the treated legs suffered a recurrence of the bioluminescence on succeeding days [106].

Another interesting contribution concerning the PDI efficiency of pL-C*e6* conjugate to treat burn infections was reported in 2009 by the same group [100]. The authors used a clinical multidrug-resistant (MDR) isolate of *Acinetobacter baumannii* genetically transformed with a plasmid encoded with the entire lux operon from *Photorhabdus luminescens* that allowed bioluminescence imaging to monitor the infection, non-invasively, in real-time. In this study, pL-C*e6* was topically applied, followed by irradiation of the burned surface with red light (660 nm) at an irradiance of 100 mW/cm^2^ and a total light dose of 240 J/cm^2^. The results showed that PDI carried out on day 0 immediately after the bacteria inoculation caused a loss of bacterial luminescence (−3 log units) in a light exposure-dependent manner. PDI carried out on days 1 and 2 reduced bacterial viability in approximately 1.7 log. It was also found that PDI did not lead to the inhibition of wound healing. Although a modest regrowth of the bioluminescent *A. baumannii* was observed in the treated burn wounds, the authors suggested that PDI may be an effective approach to treat localized infections caused by the MDR *A. baumannii* bacterium.

**Figure 20:**
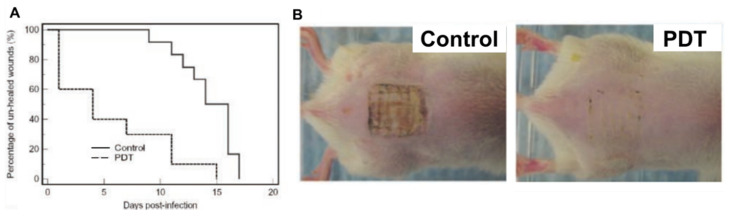
(**A**) Healing curves of MRSA infected mouse abrasion wounds without treatment and treated with PDI, respectively, and (**B**) wound morphologies at day 4 post-infection of a representative PDI-treated mouse wound (PDI) and a non-treated mouse wound (control) (figure reused from [101] with permission from John Wiley and Sons).

The PS efficacy of C*e6* covalently linked to a cross-linked polyethyleneimine (PEI) of high molecular weight (10,000–25,000) was also evaluated to treat skin wounds infected by MRSA [101]. In this work, also from Hamblin’s group, a bioluminescent MRSA strain, was used to infect wounds caused by skin abrasion in a mouse model. The PEI-C*e6* conjugate was topically applied in the infected wounds and irradiated with red light (660 nm) at an irradiance of 100 mW/cm^2^ and a total light dose of 360 J/cm^2^. The results revealed that PDI induced an average of 2.7 log of inactivation, as judged by the loss of bioluminescence in the infected wounds. It was also observed that PDI significantly reduced the bacterial bioburden in mice wounds and was able to accelerate the wound healing, when compared with untreated infected wounds (Figure 20). This fact was justified by the migration of keratinocytes from the non-wounded surrounding healthy tissue after bacteria elimination, accelerating the healing process. These promising results led authors to conclude that PDI may represent an alternative approach to treat MRSA skin infections.

**Figure 21:**
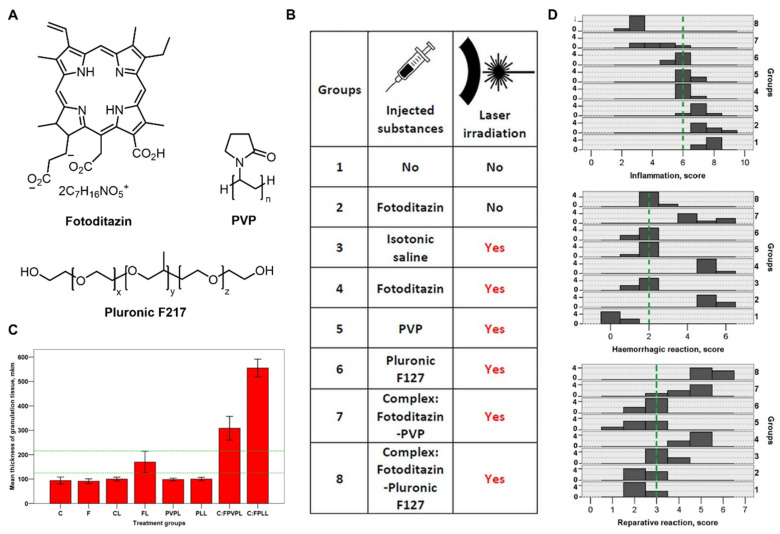
(**A**) Chemical structures of Fotoditazin, PVP and Pluronic 127; (**B**) list of the different testing groups for this experiment; (**C**) thickness of granulation tissue in the studied groups of animals; green dashed lines delineate the low and upper boundaries of CI 95% for the median value in the control group; (**D**) histograms of observed frequencies of values of morphological scores in studied groups of animals; green dashed line shows median value for all groups. (figure adapted from [107] with permission from John Wiley and Sons).

In 2014, Rudenko et al. addressed the effects of PDT on an early stage of wound healing, using two polymeric complexes containing the PS Fotoditazin (F) [107]. Fotoditazin^®^ is a commercially available version of C*e6* appearing in the form of bis-*N*-methyl-glucamine salt. The authors selected polyvinylpyrrolidone (PVP) and Pluronic F127 (PF) (a triblock copolymer based on ethylene oxide and propylene oxide) for its incorporation (Figure 21A). The biological potential of these polymeric complexes was performed in wounds created in mice and the results were compared with the ones obtained from the adequate controls (Figure 21B). All wounds were treated with red laser light (661 nm) at an irradiance of 11 mW and a total light dose of 1.45 J/cm^2^ and the procedure was repeated within three days after wounding. The results after a monitorization of four days suggested a high content of newly formed granulation tissue in the group of wounds treated with light in presence of the complex PS/pluronic F127 (FPLL), followed by the PS/PVP (FPVPL) (Figure 21C). Allied to the histological results, the authors also concluded that Fotoditazin–Pluronic F127 complex combined with light (FPLL) contributed to weaken the inflammatory processes and intensify the reparation, when compared with the Fotoditazin–PVP complex (FPVPL) and showed a significantly faster healing rate than the other groups (Figure 21D). This study also allowed to confirm the positive effect of combining polymers and PS to improve the healing process.

**Figure 22:**
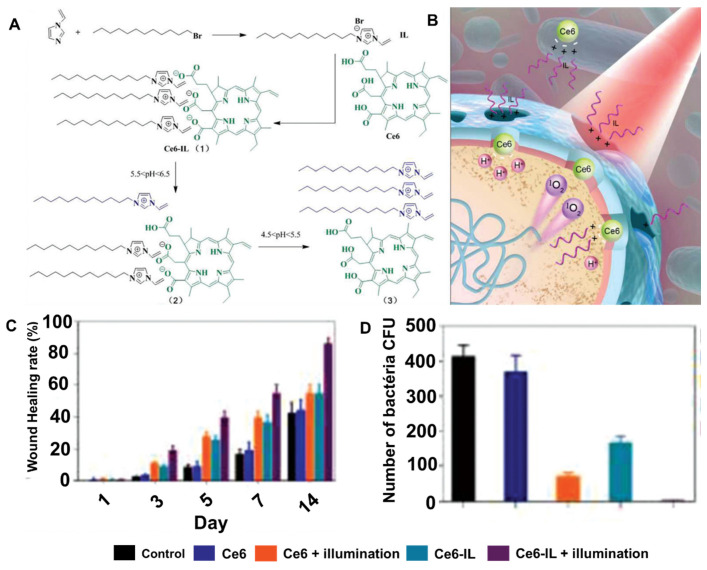
(**A**) Synthetic route for C*e6*-IL and (**B**) dual-mode antibacterial mechanism of C*e6*-IL; (**C**) wound healing rate for the different treatments 1–14 days; and (**D**) number of colonies forming units for the different treatments at day 7 (figure adapted from [108] with permission from Royal Society of Chemistry).

More recently, in 2019, Wang et al. reported an improvement in the photodynamic efficacy of C*e6* after its conjugation with the ionic liquid (IL) 1-vinyl-3-dodecylimidazolium bromide ([VC_12_Im][Br]) against *E. coli* and *S. aureus* in vitro, but also in vivo studies using infected wounds created in rabbits (Figure 22A) [108]. With the design of this assembly, the authors found that the high binding energy of the cation 1-vinyl-3-dodecyl imidazole [VC_12_Im] towards peptidoglycan (the major constituent of the cell wall) and the response of C*e6* to the acidic microenvironment of bacterial infection facilitated the PS entrance in the bacteria (Figure 22B) leading to an improvement of its photobactericidal/healing effect when compared with the corresponding controls. In fact, the results obtained with the conjugate C*e6*-IL after an incubation period of 10 min, followed by red light (660 nm) irradiation at an irradiance of 500 mW/cm^2^ for 15 min, showed a significant improvement in the healing rate after a monitorization of 14 days when compared with non-treated wounds, wounds treated with C*e6* in the absence and presence of red light, and wounds treated with C*e6*-IL in the absence of red light (Figure 22C). Moreover, the lowest bacterial load was observed in the wounds treated with the conjugate C*e6*-IL and red light (Figure 22D). 

The authors suggested that the dual mode of the antibacterial action mediated by the cation [VC_12_Im] and anion C*e6* is mainly responsible for these positive results concerning biocompatibility and antimicrobial eradication. It must be emphasized that this type of assembly–PS/IL–can be a simple approach to develop efficient antibacterial agents able to fight wound infections and promote wound healing.

**Figure 23:**
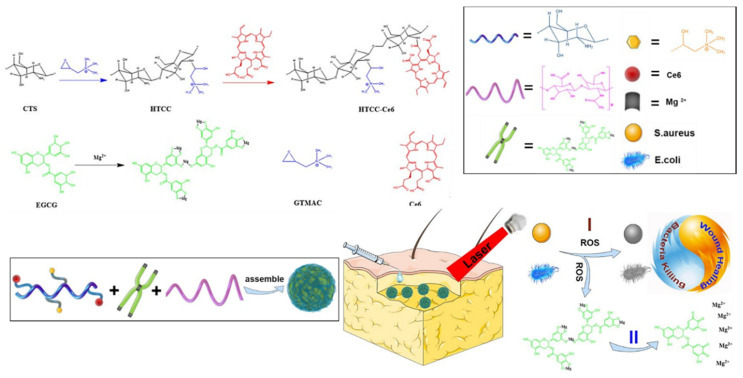
Schematic illustration for the formation of nanoparticles, starting in the synthesis of HTCC-Ce6 and Mg/EGCG complex (figure adapted with permission from [109]. Copyright (2019) American Chemical Society).

In the same year, Hu et al. selected C*e6* to design and prepare the new multifunctional quaternized chitosan (CS) complex HTCC-C*e6*–Mg/(–)-epigallocatechin-3-gallate (EGCG) (Figure 23) to treat infected wounds [109]. The synthetic strategy involved the functionalization of the CS backbone with the quaternary ammonium salt (via the epoxide glycidyltrimethylammonium chloride (GTMAC) and with C*e6* (via amide bond), affording the positively charged molecules HTCC-C*e6*. Then, these molecules were combined with Mg-EGCG complex and negatively charged hyaluronic acid molecules, affording the desired light-responsive multifunctional nanoparticles (Figure 23). With this design, it was expected that, upon contact with the ROS, the combination of C*e6*/CS bactericidal potential with the degradation response of magnesium complex and hyaluronate would induce an acceleration in the wound healing. The biological assays were performed in rats with infected wounds, using the following test’s groups: (1) non-treated wounds; (2) wounds treated only with red (660 nm) laser light at an irradiance of 100 mW/cm^2^ for 10 min; (3) wounds treated with PS nanoparticles in the absence of light; and (4) wounds treated with PS nanoparticles and red laser light. The treatments were carried out every day for 14 days. The results showed that after 7 days, the wound area in group 4 was universally smaller than the one in other groups, and, after 14 days, the wounds in groups 2 and 4 were completely healed. Contrarily, the wounds of groups 1 and 3 were not completely healed. With these results, the authors believe that this strategy offered an effective antibacterial therapeutic modality for the treatment of infected wounds, which also opened a new window to exploit promising antibacterial nanomaterials to combat antimicrobial resistance.

### 3.3. Chlorin p6

**Figure 24:**
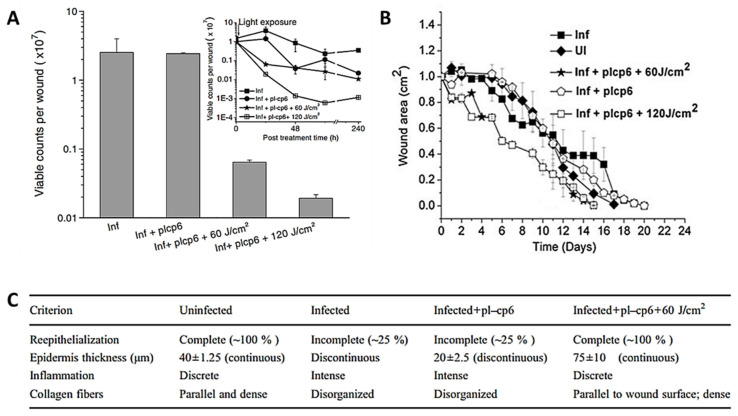
(**A**) Bacterial counts from untreated and PDI treated wounds with pL–C*p6* (=“pl-cp6”) at 24 h post-treatment; (**B**) wound area reduction of different groups as a function of time; (**C**) histomorphometric analysis of photodynamically treated and untreated wounds (figure reused from [110] with permission from Springer Nature).

Chlorin *p6* (C*p6*), also obtained from chlorophyll *a* (Figure 15), was selected by the Gupta group to develop the conjugate pL–C*p6* (analog to the Hamblin conjugate pL-C*e6*) [110]. The healing efficacy of this conjugate was evaluated in excisional wounds induced in mice and infected by *P. aeruginosa* (Figure 24). The results obtained with the infected wounds (inoculated with the bacteria 18 h before) treated with the pL–C*p6* and red light (660 ± 25 nm) at an irradiance of 100 mW/cm^2^ for 10 and 20 min (total light doses of 60 and 120 J/cm^2^, respectively) were compared with ones obtained from the following controls: (1) uninfected wounds (UI); (2) infected wounds with no treatment (Inf); (3) wounds treated only with the pL–C*p6*. The results revealed that the groups treated with pL–C*p6* and red light were the fastest to heal, comparatively to the controls (Figure 24A,B), and after 14 days these wounds were completely closed, such as the non-infected wounds. The histomorphometric analysis showed also that only these two groups were able to reach complete reepithelization (Figure 24C). The activities of important inflammatory components (cytokines IL-6, TNF-α and protease) were also monitored, and a significant reduction was observed compared to the controls. Considering that the overexpression of proinflammatory cytokine-like TNF-α is implicated in delayed healing, the results suggest that PDI, at least in part, improved the healing by reducing the hyperinflammatory conditions. Moreover, this decrease in IL-6 and TNF-α is in line with the formation of dense collagen fibers in the wounds treated with PDI after 14 days (Figure 24C). However, when compared with the uninfected wounds, there were no significant differences in the results, besides the reduced time to close the wound completely (Figure 24B).

The same group reported a posterior study, using the same PS conjugate (pL–C*p6*), where the conclusions were very similar [111]. The authors observed no differences after 14 days, when compared to uninfected wounds and murine excisional wounds infected with MRSA, and also by *P.* *aeruginosa* treated with PDI. However, from the histological analysis, it was concluded that PDI benefits wound repair in bacteria-infected wounds by significantly enhancing epithelial layer migration, hydroxyproline content, and collagen fibril arrangement. The levels of metalloproteases MMP-8 and MMP-9 (collagen degrading metallopeptidases) were also monitored, and the low levels observed for the PDI treated wounds suggest that there was an induction of the collagen remodeling, since the overexpression of these MMP is responsible for delaying collagen restoration. 

### 3.4. Sinoporphyrin Sodium

**Figure 25:**
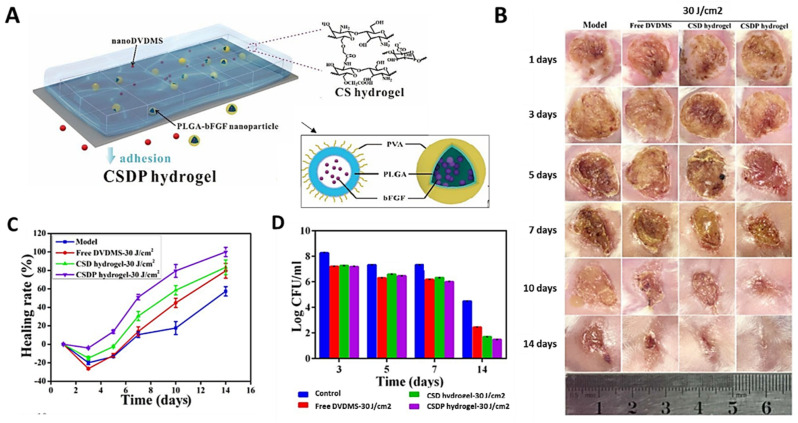
(**A**) Schematic illustration of multifunctional CSDP hydrogel; (**B**) Photographs of *S. aureus* induced infections in burn wounds obtained during the study at days 1, 3, 5, 7, 10, 14; (**C**) Measurement of wound size as shown as percentage normalized to that of the initial wound size at day 0; (**D**) bacteria counts of MRSA with CSDP hydrogel treatment at different times after treatment and of the controls (figure adapted with permission from [112]. Copyright (2020) American Chemical Society).

In 2020, Mai et al. reported the development of the photoactive CSDP hydrogel (Figure 25) using as PS the sinoporphyrin sodium (DVDMS) (structure in Figure 15) combined with PLGA-encapsulated bFGF nanospheres embedded in carboxymethyl chitosan/sodium alginate (CSD hydrogel) [112]. The antibacterial and regenerative properties of the prepared CSDP hydrogel were evaluated under irradiation and a total light dose of 30 J/cm^2^ using MDR *S. aureus*-infected burn wounds created in rats. The results obtained were compared with the ones obtained from non-treated wounds, wounds treated with the CSD hydrogel and light, and wounds treated with free DVDMS and light (30 J/cm^2^). The in vivo results obtained after monitoring 14 days revealed that the hydrogel CSDP augmented wound healing along with PDI by effective inhibition of bacterial growth, controlled inflammation, high collagen deposition, and rapid epithelialization, when compared with the other controls.

## 4. Synthetic Tetrapyrrolic Macrocycles 

### 4.1. Porphyrins and Chlorins

**Figure 26:**
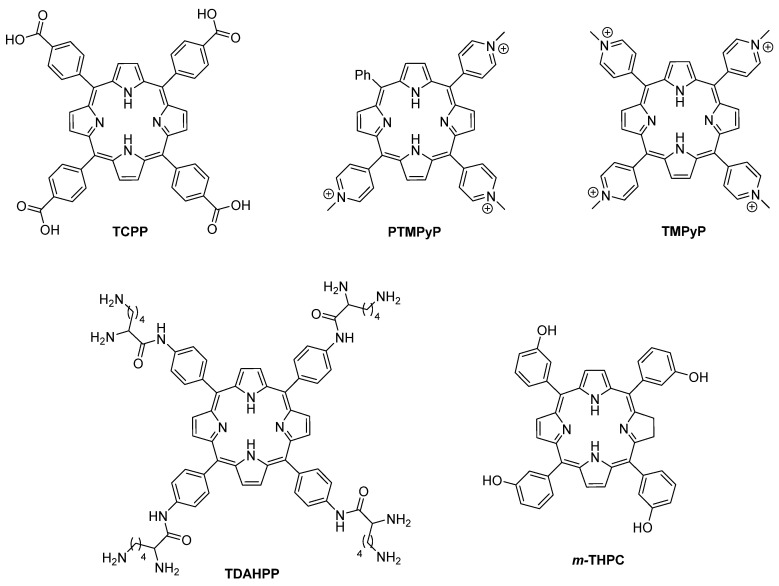
Structure of porphyrins and chlorins tested for the PDI treatment of skin wounds.

Most of the studies concerning the use of synthetic porphyrins or analogs like chlorins are based on macrocycles substituted at their *meso* positions by aryl or pyridyl groups (Figure 26). These macrocycles can be obtained by recurring to the condensation of pyrrole with appropriate aldehydes under well-established conditions [2,3]. In some cases, simple post-functionalization of those *meso*-substituents can lead to structures with high PDI efficiencies.

**Figure 27:**
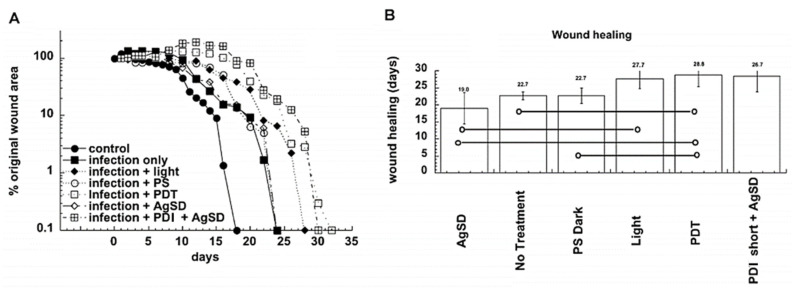
(**A**) Burn wound healing course of the burn wounds: no infection (*n* = 3); infected no treatment (*n* = 3); infected + PTMPyP without illumination (*n* = 3); infected no PTMPyP with illumination (*n* = 3); infected + topical silver sulfadiazine cream (AgSD) (*n* = 3); infected + PDI (*n* = 5); infected + PDI + topical silver sulfadiazine (*n* = 3). Illumination parameters: 635 ± 15 nm, 84 mW/cm^2^. For clarity reasons the error bars were omitted; (**B**) complete burn wound healing in days. Lines connect the groups that significantly (*p* < 0.05) differ from each other (figure reused from [113] with permission of Royal Society of Chemistry).

In 2005, the group of Hamblin evaluated the in vivo efficacy of 5-phenyl-10,15,20-tris(1-methylpyridinium-4-yl)porphyrin chloride (PTMPyP) (Figure 26) as PS to treat 3rd degree burn wounds infected with bioluminescent *S. aureus* (Figure 27) [113]. The PDI assays were performed 24 h after the *S. aureus* infection under red light (635 nm) irradiation at an irradiance of 84 mW/cm^2^)^.^ and in the presence of PTMPyP at 500 μM. A sensitive ICDD camera was used to monitor the evolution of the wounds through bioluminescence imaging. The results were compared with the ones achieved using a silver sulfadiazine cream (AgSD), a standard clinical treatment for infected burns. The results showed that more than 98% of the bacteria were photoinactivated after receiving a total light dose of 210 J/cm^2^. Moreover, the PDI mediated a faster reduction of bacterial concentration in the burn wound (it occurs in less than 1 h) than the bacterial reduction achieved with the standard silver sulfadiazine cream approach, which takes several days to occur. However, bacterial re-growth after PDI treatment was observed and the wound healing was delayed by PDI or just by the application of light regime. Nevertheless, the authors highlighted that PDI treatment must be optimized by improving the PTMPyP affinity to the bacteria to prevent their re-growth and to exert a tissue-sparing effect. It was recognized that an optimization of the PS absorption coefficient in the red region of the electromagnetic spectrum should also be considered [113].

**Figure 28:**
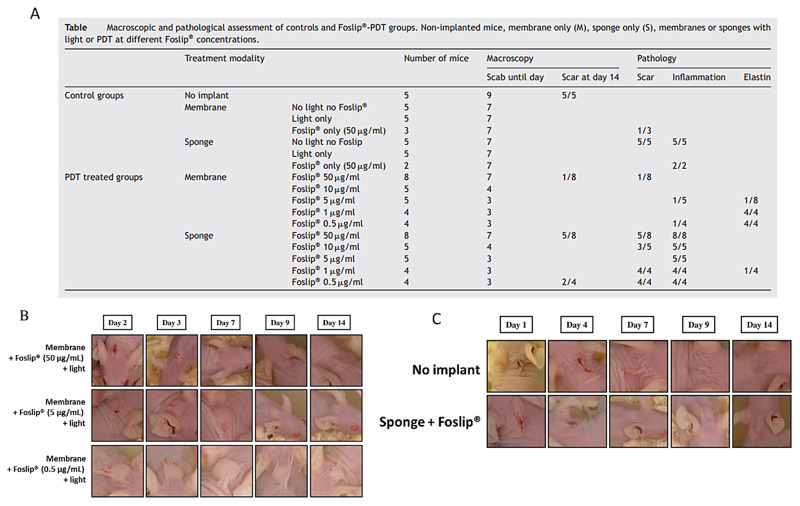
(**A**) Different groups tested in this study; (**B**) macroscopic assessment of wound healing during 14 days for the groups treated with Foslip^®^ followed by red light irradiation; and (**C**) controls (figure reused from [114] with permission of Elsevier).

In 2011, the group of D’Hallewin compared the effect in skin wound healing of two collagen-based scaffolds embedded in a Foslip^®^ formulation [114]. This formulation contains 5,10,15,20-tetrakis(3-hydroxyphenyl)chlorin (Foscan^®^, *m*-THPC), a PS clinically approved for the palliative treatment of head and neck cancers, in dipalmitoylphosphatidylcholine/dipalmitoylphosphatidylglycerol liposomes. The selection of collagen-based scaffolding materials (a BioGide membrane and a Kollagen Resorb sponge) was based on their efficiency to restore skin integrity due to their biocompatibility and non-toxic features allowing the cellular attachment, growth, and differentiation without excessive scar formation. In this study, the authors used sponges or membranes incubated with different concentrations of Foslip^®^ (50 g/mL, 10 g/mL, 5.0 g/mL, 1.0 g/mL or 0.5 g/mL) to implant in clear-cut incisions in mice, which were then sutured. The results obtained after red laser irradiation (Ar-pumped dye laser at 652 nm, at an irradiance of 100 mW/cm^2^ and a total light dose of 10 J/cm^2^) were compared with two controls: injuries with no treatment and injuries implanted with the sponge scaffold in the absence of light irradiation (Figure 28). The injuries were monitored for 14 days, and the authors observed that the photodynamic treatment at the three lowest concentrations (0.5, 1.0 and 5.0 g/mL) had a positive impact on wound healing. The scab detachment was observed on the 3rd day, while in the other groups, at the 7th day or after. Inflammation and progressive resorption of the implant material are more pronounced in those categories. The study showed that scarring is more frequent in the sponges rather than in the membranes. The authors believe that this can be attributed to the presence of exogenous elastin in the membrane complexes, which might stimulate the final matrix reorganization. Elastin neosynthesized, indicating the final stages of wound healing, is only found when the photodynamic treatment is applied at the three lowest concentrations and is more intense in the presence of membranes as opposed to sponges. However, the authors suggested that further investigations must be carried out to establish the mechanistic aspect of improved wound healing with low dose Foslip^®^ followed by light treatment (PDT treated groups). For that, they concluded that future identification and quantification of the cytokines involved can offer hindsight to understand how the treatment influences inflammation, which is an essential early-stage process to induce wound healing.

**Figure 29:**
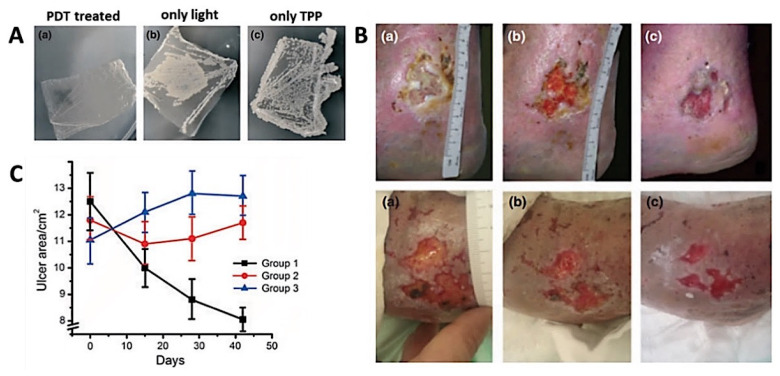
(**A**) Antimicrobial effect of nanofiber textiles (NTs) in different treatment modalities against *S. aureus*; (**B**) clinical pictures of two patients who were treated with illuminated, TPP-doped nanofiber textiles at baseline (a,a), days 15 (b,b) and 42 (c,c) of treatment; (**C**) changes in ulcer surface areas (figure reused from [115] with permission of John Wiley and Sons).

In 2012, Arenbergerova et al. evaluated the antibacterial effect of electrospun polyurethane nanotubes doped with 5,10,15,20-tetraphenylporphyrin (TPP) (Figure 29) in the treatment of patients with leg ulcers under PDI conditions (white light irradiation with a 150 W halogen light) [115]. These patients, designed as Group 1, received treatment for six weeks and the healing efficacy was monitored and compared with ulcers that were irradiated just in the presence of polyurethane nanotubes (Group 2) and with ulcers where the patients were treated with nanotubes doped with TPP in the absence of light (Group 3) (Figure 29A). It was observed that all groups presented an improvement of the wounds, but the wounds from Group 1 presented a more significant area reduction when compared to the other testing groups (Figure 29B,C). The authors believed that this PDI approach represented a suitable alternative to the use of topical antibiotics and antiseptics, and also has the potential to be explored in regenerative medicine.

**Figure 30:**
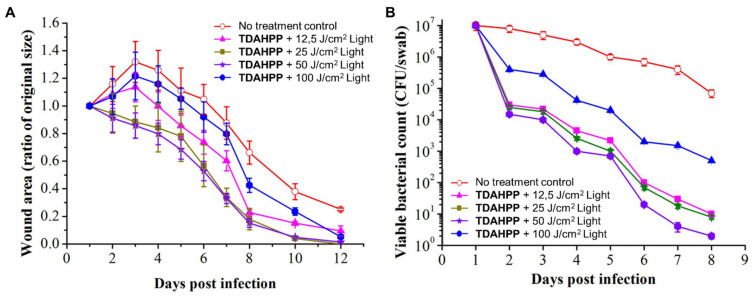
(**A**) Wound healing ratio over time in bacterial infected rats with and without TDAHPP; (**B**) mean bacterial counts of the swabs collected from the rat wounds during the eight days post-infection (figure adapted from [116] under Creative Commons Attribution License).

The effect of 5,10,15,20-tetrakis(4-((*S*)-2,6-diaminohexanamido)phenyl)porphyrin (TDAHPP, Figure 26) after irradiation with red light (650 nm; at an irradiance of 100 mW/cm^2^) and using different light doses in the treatment of rats infected wounds by mixed bacteria (MRSA, *E. coli* and *P. aeruginosa*) was evaluated by Liu and co-workers in 2016 [116]. The assays were performed using (A) non-treated wounds; (B) wounds treated with TDAHPP and irradiated with red light for total light dose of 100 J/cm^2^; (C) wounds treated with the TDAHPP and irradiated with red light for a total light dose of 50 J/cm^2^; (D) wounds treated with the TDAHPP and irradiated with red light for a total light dose of 25 J/cm^2^; and (E) wounds treated with the PS and irradiated with red light for a total light dose of 12.5 J/cm^2^. The wounds were treated three times and monitored for 12 days. All the wounds revealed a decrease in size throughout time. However, the wound healing ratio is in the order of C > D > E > B (Figure 30A). The antibacterial activity of the PDI treatment was also analyzed (Figure 30B) and the results indicated that the wounds treated under the PDI procedure suffered a proportional bactericidal effect regarding the light dose used. The results showed that this PDI treatment not only leads to bacteria photoinactivation but also improved tissue repair. The best results were obtained using total light doses between 25 and 50 J/cm^2^.

In the same year, Fila et al. have reported the development of a novel murine model exhibiting the key hallmarks of chronic wounds, based on full-thickness skin wounds paired with an optically transparent cover to study aPDT using 5,10,15,20-tetrakis(1-methylpyridinium-4-yl)porphyrin (TMPyP) (Figure 26) as well other 3 non-porphyrinic PS [Rose Bengal (RB), New Methylene Blue (NMB) and [Ru(2,2′-bipyridine)2(2-(2′,2″:5″,2‴-terthiophene)-imidazo [4,5-f][1,10]phenantroline]^2+^ (TLD141)] [117]. MRSA (XEN) and *P. aeruginosa* (PAK) were the two bioluminescent pathogens used. PAK is a wild type, commonly studied *P. aeruginosa* strain that contains and expresses a full complement of virulence factors. The porphyrinic derivative TMPyP, as well all the 3 non-porphyrinic PS, revealed to be excellent in the bacterial inactivation of planktonic cells. For PAK, a 6 log of photoinactivation was achieved with TMPyP under LED irradiation (525 nm) with a total light dose of 150 J/cm^2^, which is considered a sterilized state. However, the high in vitro efficacy of this PS was poorly translated into in vivo applications. In this case, the growth delay was limited with 24–48 h delay for MRSA; it was also noticed that there was a longer growth suppression of *P. aeruginosa* with TLD1411 mediated by PDI. This was explained by considering host factors as well as deviating bacterial burdens, and the virulence of individual strains that represented a more challenging environment for the treatment of human superficial infections [99]. Nevertheless, the authors admitted that the significant clinical reduction of bacterial concentration remains feasible, causing a delay in the appearance of a fully activated inflammatory response, thereby reducing the risk of developing sepsis [117].

**Figure 31:**
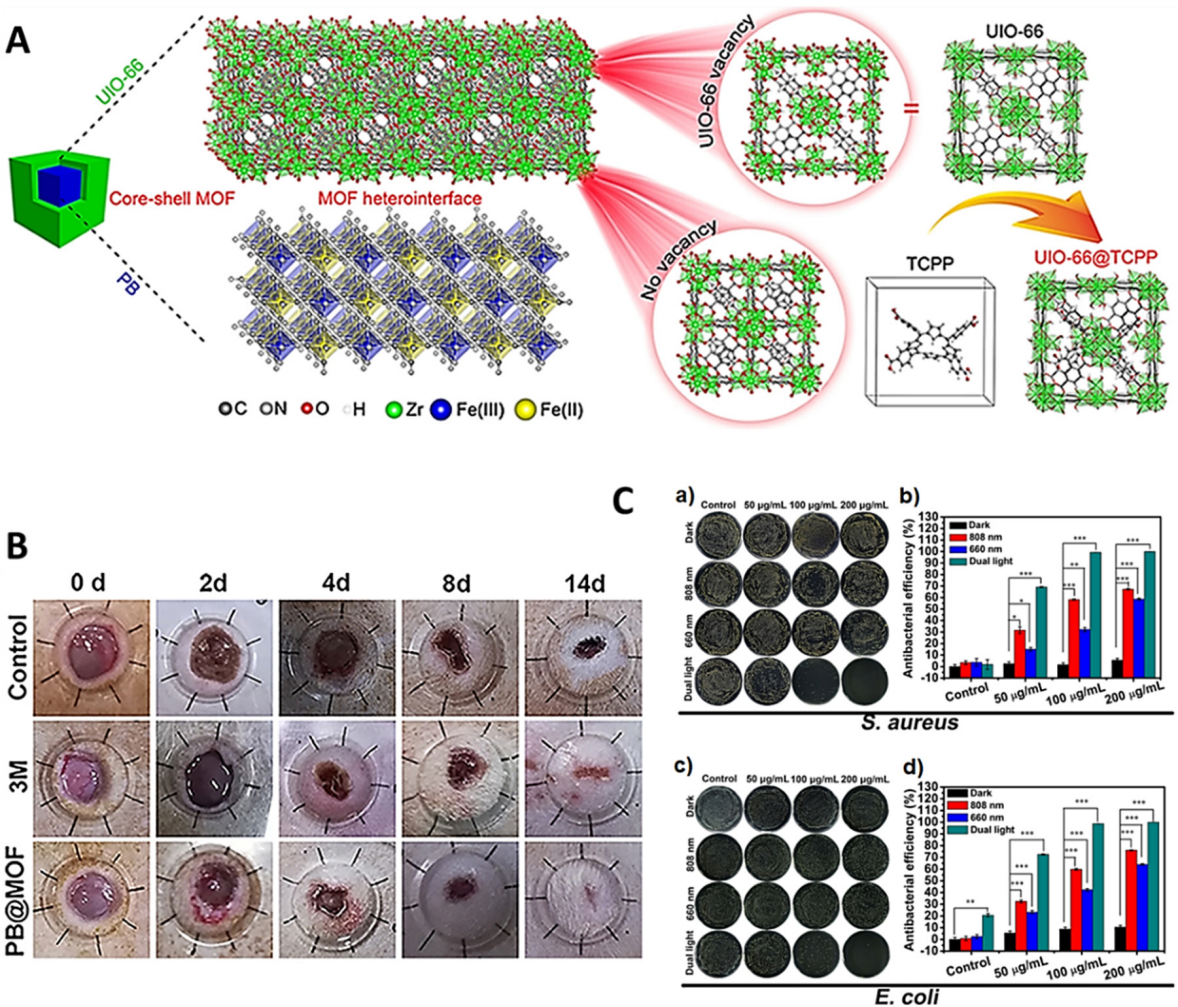
(**A**) Schematic illustration of the core−shell structure of PB@MOF; (**B**) photographs of a wound in the group of control, 3M, and PB@MOF at different points of 0, 2, 8, and 14 days; (**C**) photographs of bacterial colonies formed by (**a**) *S. aureus* and (**c**) *E. coli* and corresponding antibacterial rates of (**b**) *S. aureus;* and (**d**) *E. coli* under the conditions of darkness and after exposure to 808 nm NIR, 660 nm red light, and to dual light for 10 min (figure reused with permission from [118]. Copyrighted (2019) American Chemical Society). * *p* < 0.05; ** *p* < 0.01; *** *p* < 0.001.

Luo and co-workers explored the use of photoactive metal-organic frameworks, (MOF) in wounds infected with *S. aureus* or *E. coli*, created in rats [118]. The structures (Figure 31A) were obtained using the FDA-approved Prussian Blue MOF (PB@MOF) as the core, and 5,10,15,20-tetrakis(4-carboxyphenyl)porphyrin (TCPP) (Figure 26) as the shell. After application of MOF in the wounds, they were irradiated under dual light, 808 nm (NIR, 500 mW/cm^2^) plus 660 nm (red light, 78.5 mW/cm^2^) for 10 min. Wounds treated only with PBS or with the commercially available wound dressing 3M were used as controls. The follow-up showed that in contrast to the control and 3M groups, the best wound healing rate was obtained under the dual light illumination group, allowing the wound to be entirely healed after 14 days (Figure 31B). The antibacterial effect of PDI treatment under dual light irradiation was also compared with the outcomes obtained when the irradiations were performed using only a single light at 808 nm or 660 nm (Figure 31C). The most effective killing of both *S. aureus* and *E. coli* (more than 99%, 2 log of reduction in bacterial abundance) was observed for the MOF under dual light irradiation.

**Figure 32:**
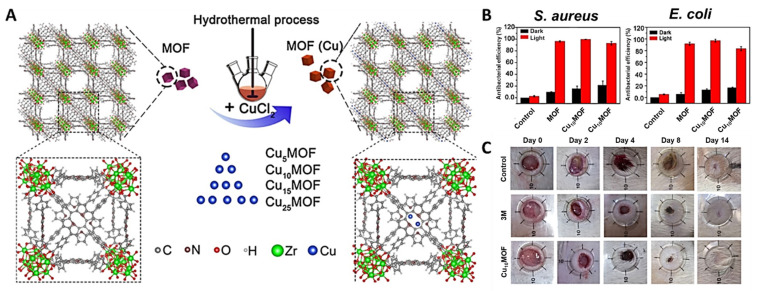
(**A**) Schematic illustration of the preparation procedures of different kinds of MOF, as well as the crystal structure, structural components, and underlying network topology of the obtained MOF; (**B**) results of the corresponding antibacterial activity; (**C**) in vivo assessments of treatment of the samples (PBS, 3M wound dressing, and Cu_10_MOF) for wound healing (figure reused from [119] with permission from Elsevier).

In 2020, the studies were extended to another porphyrinic MOF referred to as PCN-224 obtained through the connection between Zr_6_ clusters and TCPP but doped with different amounts of Cu^2+^ [119]. The Cu-dopped MOF nanoparticles Cu_5_MOF, Cu_10_MOF, Cu_15_MOF, Cu_25_MOF were obtained by reacting PCN-224 with adequate amounts of CuCl_2_ using a hydrothermal approach (Figure 32A). The biological assays showed that the framework Cu_10_MOF presented the best in vitro antibacterial performance against *S. aureus* (99.71%) and *E. coli* (97.14%) after 20 min of red-light irradiation at 660 nm (Figure 32B), and consequently was selected for the in vivo PDI assays carried out in mice wounds with the bacteria (Figure 32C). The results obtained with Cu_10_MOF after being irradiated with red light at an irradiance of 400 mW/cm^2^ for 20 min were compared with the ones obtained from wounds treated only with PBS and wounds treated with the traditional 3M wounds dressing. A follow-up after 14 days showed an acceleration in wound healing, compared to the controls in the wounds subjected to PDI mediated by MOF (Figure 32C), without showing any appreciable toxicity. This effect was more notorious at the earlier stage, which could be explained by the release of Cu^2+^, as also shown in previous results [120]. These results open new application perspectives for MOF under the context of wound healing, especially when the wound is infected with bacterial strains.

Analogous results were attained when the same approach was applied by using a MOF prepared with TCPP as the ligand doped with dopamine. The deposition of dopamine at the MOF surface increases its absorption at 660 nm leading to an enhanced photothermal and photocatalytic activity against *E. coli* (99.97%) and *S. aureus* (99.62%). Even in vivo, when irradiated with red light (650 nm) at an irradiance of 700 mW/cm^2^ for 20 min, the MOF-porphyrin-based PS doped with dopamine exhibited a distinguishable capability to eliminate bacterial infections with a concomitant increase in skin wound healing process when compared with the control groups [121].

**Figure 33:**
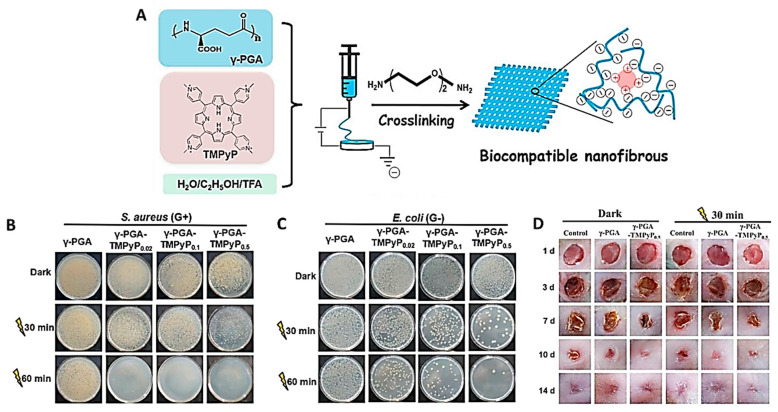
(**A**) Schematic illustration of the fabrication of poly(γ-glutamic acid)/TMPyP nanofibrous mat with photodynamic antibacterial activity; LB-agar plates photographs of residual (**B**) *S. aureus* and (**C**) *E. coli* on various nanofibrous mats under different antibacterial treatments (dark, 30 min and 60 min light irradiations); (**D**) typical photographs of the wounds at 1, 3, 7, 10, and 14 days after different treatments (figure reused from [122] with permission of Elsevier).

Sun and coworkers reported a study concerning the application of a PS nanofibrous mat composed of Poly(γ-glutamic acid) (γ-PGA) and the porphyrin TMPyP (Figure 33A) to treat rat infected wounds [122]. The lesions were monitored for 14 days after irradiation periods of 30 min and 60 min, and the results revealed that the materials have good biocompatibility, which was attributed to the moist environment at the wound bed due to the γ-PGA hydrophilic features. Antimicrobial in vitro studies against *E. coli* and *S. aureus* revealed that the PDI treatments had a positive effect on decreasing the bacterial load (Figure 33B,C). Furthermore, the authors observed that the PDI treatment using the photosensitizing polymeric nanofibrous mat can decrease the inflammatory phase and accelerates wound healing with negligible local toxicities (Figure 33D). These results led the authors to conclude that these rated TMPyP nanofibrous mats may show great potential in antibacterial wound dressing applications.

### 4.2. Phthalocyanines

**Figure 34:**
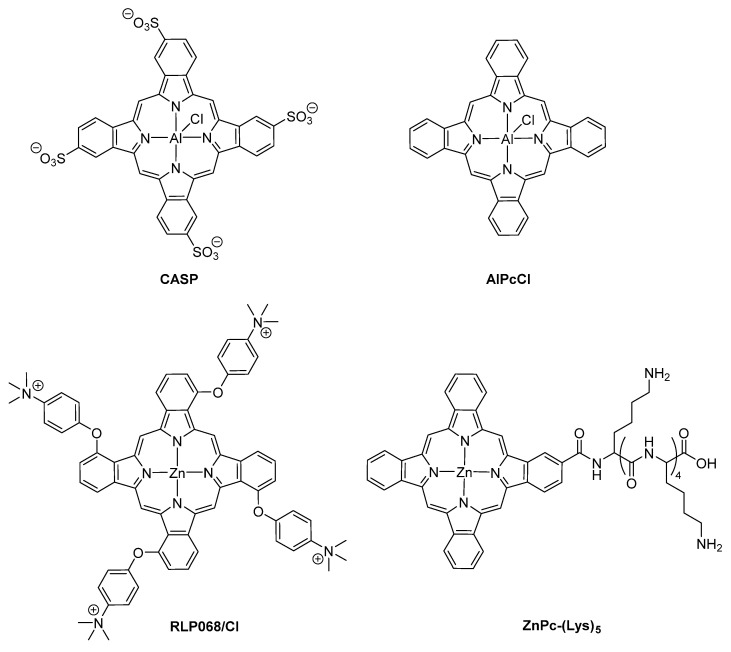
Chemical structures of phthalocyanines used as PS in wound healing.

Other tetrapyrrolic macrocycles that are meriting the attention from the scientific community as PS for skin wound healing are phthalocyanines (Pc). These synthetic molecules (Figure 34) present a highly intense Q band at ca. 700 nm allowing their activation with red light, which can cross tissues far beyond 6 mm. This is particularly relevant for treating local skin infections that are deep-sited or in the lining of internal organs using adequate light medical devices (e.g., light guide lasers). These macrocycles are, in general, obtained by condensation of proper precursors, such as phthalonitriles, 2-cyanobenzamide, phthalimide, diiminoisoindoline or phthalic acid, and phthalic anhydride at high temperatures (>150 °C) and in the presence of a metal or metal salt [123]. The introduction of adequate substituents at the peripheral positions of the tetraazaisoindole macrocycles or their embedment/immobilization in adequate supports or nanocarriers can improve the Pc photodynamic efficacy due to their general high tendency to aggregate, either in organic and aqueous solutions.

In 1990, Stern and coworkers evaluated the effect of the PDI treatment mediated by chloroaluminum sulfonated phthalocyanine (CASP) (Figure 34) in wounds created in rats [124]. In this study, the results obtained from the PDI treatment (CASP irradiated with light at 675 nm; 200 mW/cm^2^, 100 J/cm^2^) indicated a direct effect on the neovasculature of the healing wounds, when compared with controls (non-treated wounds and wounds subjected to the same irradiation protocol in the absence of the PS). However, it was emphasized that further studies were necessary to clarify the nature and the time course of vascular events occurring after PDI with CASP.

Less positive achievements were reported in 1999 by Pareck and coworkers when the healing effects of CASP and benzoporphyrin mono-carboxylic acid (BPD-MA, Figure 15) (obtained from PP-IX and commercialized as Visudyne^®^) were compared in rat wounds [125]. In these in vivo assays, CASP was administered to the wounds in concentrations of 0.25 mg/kg or 0.5 mg/kg, while BPD-MA was administered to the wounds in concentrations of 5 mg/kg or 10 mg/kg. The wounds were then irradiated using the appropriate light parameters for each PS: 690 nm (400 mW/cm^2^) for BPD-MA and 675 nm (400 mW/cm^2^) for CASP. The wounds serving as controls were left untreated. The study did not show any benefit of PDI either in the rate of healing or in scar quality when compared to the controls. Nonetheless, the authors suggested that the incisional wound healing model used in this study was insensitive to the subtle effects that may occur when PDI was mediated by BPD-MA or CASP.

In 2004, Silva et al. reported an encouraging study where the results clearly indicated a synergetic effect on tissue healing after PDI treatment mediated by the aluminum complex of phthalocyanine (AlPcCl) (Figure 34) embedded in a gel-based (GB) drug delivery constituted by the commercially available “Poloxamer 407” and “Polyetilenoglicols PEG 400” [55]. The results obtained from cutaneous wounds in rats irradiated in the presence of the PS plus gel base (685 nm, 2.5 J/cm^2^, 35 mW; LPSG) were compared with ones obtained from untreated wounds (CG) and just treated with gel base (GB); PS; light (LG); and PS + light (685 nm, 2.5 J/cm^2^, 35 mW; LPS). The treatments were performed daily using a laser InGaAIP and the wounds were monitored for seven days. The best results were obtained from the groups LPS, LPSG, and LG, when compared with the CG and GB controls. The histological analysis also revealed that the groups receiving the PS presented faster healing, with stronger collagen deposition in a more extensive extracellular matrix. Moreover, the re-epithelialization was more effective in the LPSG group and the remodeling of the connective tissue was more evident in the LPS and LPSG groups. These results showed that a low-level laser combined with a PS can improve the healing process.

**Figure 35:**
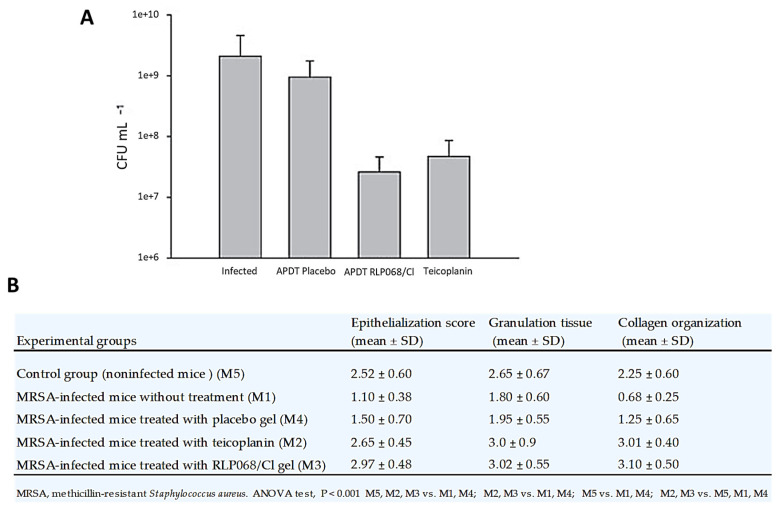
(**A**) Bacterial load in infected mice and three contaminated groups, treated, respectively, with: antimicrobial photodynamic therapy (APDT) with topically applied RLP68⁄Cl, placebo gel (no PS), and intraperitoneal teicoplanin, nine days post-infection with MRSA; (**B**) summary of the biological impact of different treatment on wound-healing parameters at day 9 from infection (figure reused from [126] with permission of John Wiley and Sons).

In 2011, Simonetti and co-workers evaluated the effect of the tetracationic Zn(II) phthalocyanine RLP068/Cl (Figure 34) using a mouse model with wounds infected with MRSA in order to mimic non-healing ulcers in humans [126]. The PS was first incorporated into a gel formulation of a carboxymethyl cellulose polymer and after being applied in the wounds was irradiated with diode laser (698 nm; 120 mW/cm^2^, 60 J/cm^2^). For controls, non-treated wounds, wounds treated with the antibiotic teicoplanin, and wounds just irradiated in the presence of placebo gel were used (Figure 35A). The results revealed that the average bacterial inactivation was slightly superior in the PDI treatment, comparatively to the wounds treated with teicoplanin. No reduction was observed in the controls of wounds not treated, or wounds that received only light. The histological comparison of the wounds from untreated versus treated mice showed the positive impact of both treatments in the wound-healing response (Figure 35B). The authors also emphasized that the re-epithelialization of the wounds with a continuous epithelial lining and overall keratinization was slightly more organized in the aPDT group than in the group treated with teicoplanin.

**Figure 36:**
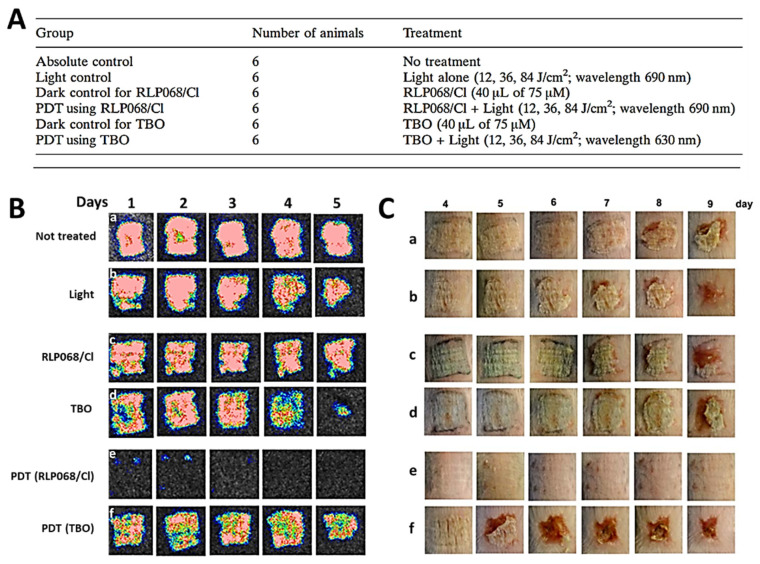
(**A**) Experimental plan of animal experiments; (**B**) (a-f) successive bioluminescence images of the follow-up of mouse scratch infected wound with MRSA, in the different groups; (**C**) macroscopic assessment of wound healing from the 4th until 9th day of different groups (figures reused from [127] with permission from John Wiley and Sons).

In 2013, Hamblin et al. evaluated the efficiency of the tetracationic zinc(II) phthalocyanine derivative RPL068/Cl (Figure 36) as the PS in the photodynamic inactivation of bioluminescent MRSA in a mouse skin abrasion model. The wound healing effect of this approach was also evaluated. The results were compared to the ones achieved with toluidine blue (TBO) [127]. The wounds were inoculated with bioluminescent MRSA and both PS were added 30 min after infection. Mice were irradiated with light at 690 nm for RPL068/Cl and 630 nm for TBO at an irradiance of 100mW/cm^2^ (Figure 36A). The results showed that the PDI treatment using RLP068/Cl was effective in the photoinactivation of MRSA (Figure 36B), inhibiting the bacterial regrowth, but also in the wound healing which occurred earlier than in the control groups (Figure 36C). The promising results with RLP068/Cl versus TBO were associated with a faster bacterial inactivation within the lesion since the presence of bacteria are known to inhibit the wound healing, although the importance in clarifying the wound healing mechanism mediated by RPL068/Cl was emphasized [127].

In 2018, Mosti and coworkers reported a promising pilot experience where aPDT mediated by RLP068/Cl was used in the treatment of infected leg ulcers of 36 patients waiting for skin grafts [128]. Although the most common bacterial species found were the Gram-positive *S. aureus* and the Gram-negative *P. aeruginosa,* some resistant bacteria to common antibiotics were also detected. The protocol involved the irradiation of the ulcers 30 min after the PS application with red light at 630 nm for 8 min (60 J/cm^2^). The protocol was repeated after 72 h and after this second treatment, the bacterial load in 32 patients (four were dismissed due to severe pain) was close to zero except in two ulcers where some bacteria were still detected. It was highlighted that clinical signs of biofilm were not visible, and the ulcers response was not dependent on their pathophysiology. Although the authors were a bit cautious due to the limited population tested and the lack of a control, it was considered that the results are in line with the previous outcomes of a previous study where the same PS was used to treat, for the first time, infected diabetic foot ulcers (DFU) [129]. However, and contrary to Mosti’s study, the previous treatment involved the combined administration of amoxicillin (875 mg) plus clavulanic acid (125 mg) three times a day, for the first seven days of the treatment. So, there were some doubts about the more notorious decrease of the bacterial load in the treatment group (PDI + antibiotic), compared to the placebo antibiotic group.

**Scheme 1:**
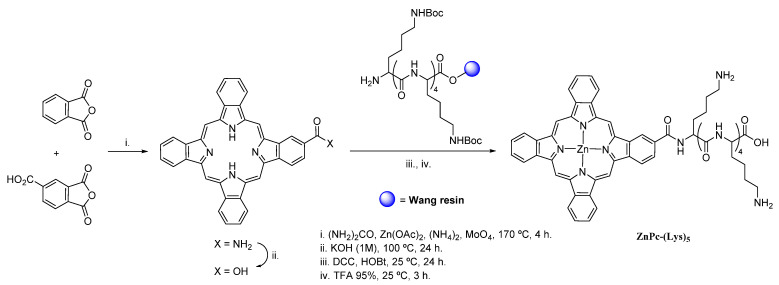
Synthetic approach to prepare ZnPc-(Lys)_5_.

Considering the high efficacy of the zinc complex of pentalysine β-carbonylphthalocyanine [ZnPc-(Lys)_5_] (Scheme 1) to photoinactivate in vitro nosocomial MDR bacteria, such as *MDR-E. coli* (MREC) and *MDR-A. baumannii* (MRAB) under red light, Wang and coworkers considered its use to treat in vivo infected wounds in mice [130]. The strategy to prepare this PS was previously reported by the authors and involved the coupling of the unsymmetrical Pc ZnPc-COOH with pentalysine in the presence of 1-ethyl-3-(3-dimethylaminopropyl)carbodiimide (EDC) and *N*-hydroxysuccinimide (NHS) (Scheme 1) [131].

**Figure 37:**
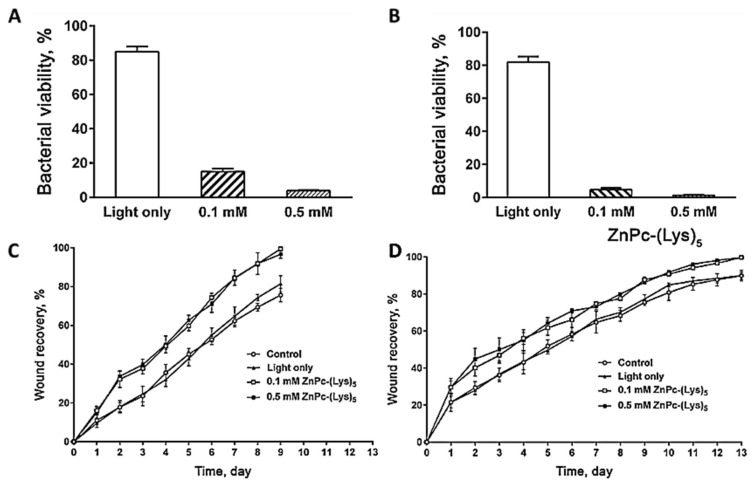
Reduction. (**A**) Multidrug-resistant *E. coli* (MREC) and (**B**) multidrug-resistant *A. baumannii* (MRAB) in infected incision wounds on mice upon aPDT treatment with ZnPc-(Lys)_5_; effects of ZnPc-(Lys)_5_ concentration on the aPDT healing of (**C**) MREC and (**D**) MRAB (figure adapted from [130] with permission from World Scientific Publishing Co., Inc., Singapore).

In the in vivo studies, the excisional wounds were inoculated with MREC or MRAB and the results obtained with the wounds treated with the PS (0.1 mM or 0.5 mM) and irradiated with LED light at 680 nm (100 J/cm^2^), were compared with the wounds treated only with LED light. The aPDT results showed a faster recovery for the MREC infected wounds than for the MRAB-infected ones (9 days *versus* 13 days) (Figure 37). This difference in the healing time was associated with a possible difference in the toxicity of different MDR strains to the skin since in vitro, the better efficacy was found for the inactivation of MRAB. In the wounds treated just with red light, the healing was around 80% for the same treatment period. The higher PS concentration required for the in vivo studies was justified by PS with nonspecific binding to the wound tissue instead of selective absorption by bacteria and by the occurrence of some photobleaching caused by the higher light dose required for an efficient in vivo treatment.

**Figure 38:**
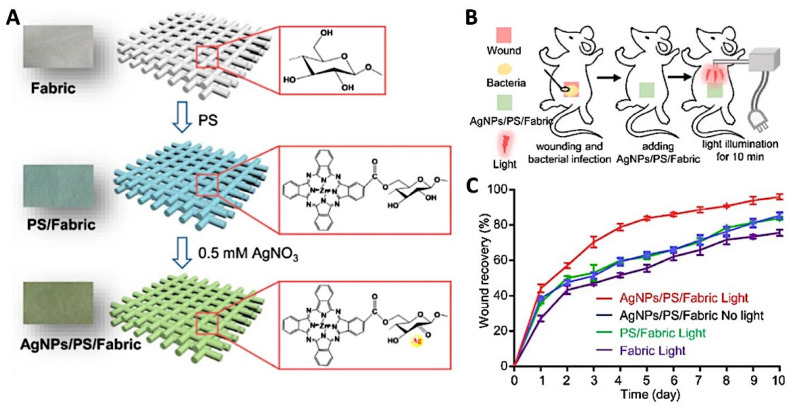
(**A**) Design of AgNP/PS/Fabric material with dual antimicrobial mechanisms; (**B**) experimental schematic diagram of bacterial infection treatment in vivo; (**C**) effects of AgNP/PS/Fabric on the healing of bioluminescent *E. coli* infected excisional wounds in mouse (figure reused from [132] with permission from Elsevier).

In 2019, Chen et al. reported that the composite AgNP/PS/fabric constituted by a cellulose fabric, the Zn(II) complex of the β-carboxyphthalocyanine (ZnPcCO_2_H) and silver nanoparticles (Figure 38A) with antimicrobial efficacy towards *E. coli*, a bioluminescent *E. coli*, *S. aureus* and MRSA bacteria was able to promote wound healing [132]. The photoactive material preparation involved the activation of the Pc carboxyl group with *N,N′*-carbonyldiimidazole in order to promote the ester covalent linkage with the cellulose fabric, followed by the silver nanoparticles preparation. These nanoparticles were obtained by reduction of silver ions mediated by the fabric during the autoclave procedure. The bacterial efficacy of the composite was justified considering the enhancement in the generation of ROS by the PS mediated by the silver nanoparticles and by the silver ions release during the photosensitizer activation. In the in vivo studies, the mice wounds inoculated with luminescent *E. coli* were covered with the AgNP/PS/fabric and irradiated with red light for 10 min (75 mW/cm^2^) (Figure 38B). The results obtained after monitoring the wound size for 10 days showed that the PDI treatment in the presence of AgNP/PS/fabric conducted to faster healing when compared with the ones obtained from the adequate controls: just irradiation; PS/fabric + irradiation; AgNP/PS/fabric in the absence of irradiation (Figure 38C). The results suggested that the combination of AgNP and PS in PDI treatment of wounds has a positive impact when compared to the use of the PS or AgNP only. Nonetheless, the treatment using PS/fabric was also considered positive and can justify the use of this PS for future studies in skin wound healing.

## 5. Final Remarks

In summary, PDI with porphyrinoids-based photosensitizers exhibit great potential for the treatment of skin wounds, inducing healing in bacteria-infected wounds. Noticeably, in recent years, significant efforts were performed by the scientific community to improve the use of tetrapyrrolic macrocycles in skin wound healing. The capability of porphyrinoids to act as efficient PS (Table 2) relies on their capability to generate ROS when irradiated at wavelengths comprised in the “therapeutic window”.

ALA and derivatives are used as the precursors of protoporphyrin-IX, increasing the PS accumulation in the target area and consequently the photodynamic process efficiency. Natural chlorin derivatives can be used solo or modified with appropriate units, such as poly-Lysine, ionic liquids, and incorporated into micelles to increase its lipophilicity. In fact, the reported studies show that the pathway to improve the efficiency of porphyrin-based PS in skin wound healing passes throughout the exploitation of using PS immobilized/supported into solid matrixes. This strategy is more advantageous since it allows to merge the features of both PS and matrix.

The use of (bio)polymers, such as chitosan, collagen, alginate, and polyurethane, allows to match the PS features with the biocompatibility, degradability, and stimulating properties of the polymer. This is a significant input that makes available the use of molecules with low solubility but remarkable ROS production, such as some synthetic *meso*-tetraarylporphyrins or phthalocyanines.

Porphyrinoids display high synthetic versatility, allowing the preparation of several different PS delivery systems in the target wound site. Tetrapyrrolic can be directly attached to the matrix through covalent bonds, encapsulated in polymeric nanocarriers, incorporated by a physical mixture with hydrogels, or used to decorate polymeric systems throughout non-covalent approaches.

The symbiotic effect of PS and biocompatible polymeric systems improve not only the healing rate, but also the PS efficiency against Gram-negative bacteria (bacteria that are less affected by PDI) growth and consequent biofilm formation, the optimization of the PS into the wound, and the PS degradation rates slow down. Moreover, this approach allows topical application at the wound site which is a significant advantage for the regenerative and skin wound healing processes.

Considering that the limited clinical success of wound healing [133] is associated with the lack of (i) funding, (ii) centers of excellence concerning wound care management, (iii) methodologic consistency in clinical trials, and (iv) the appearance of more complex pathologies due to the populations aging, and too many drugs with limited efficacies, we believe that PDI/healing can have an opportunity to overcome some of these drawbacks. This approach can improve the local asepsis due to its antimicrobial effect, which includes areas with diminished blood irrigation, and where the conventional antimicrobials do not work. Consequently, it will reduce patient pain and increase cosmetic effects due to enhanced cell proliferation. Additionally, it can be performed in ambulatory care environments, requiring low-cost light sources, and demanding easy clinical staff training. Furthermore, the treatment can be repeated without resistance development.

## Figures and Tables

**Table 1:** Wound closure patterns of different groups of animals [72].

Entry	Sample	Wound Healing Period (days) ^a^
1	Control-1	19 ± 0
2	Control-2 (ALA only)	20 ± 1
3	Control-3 (HpD only)	18 ± 2
4	Test-1 (ALA + He-Ne)	13 ± 1
5	Test-2 (ALA + He-Ne + Nd:YAG)	17 ± 1
6	Test-3 (HpD + He-Ne)	16 ± 2
7	Test-4 (HpD + He-Ne + Nd:YAG)	14 ± 1

^a^—the values given are the mean of 12 wound sites.

**Table 2:** Summary of the studies discussed in this review concerning the use of porphyrinoid PS for skin wound healing.

	Wound Type	Infection	PS	Log Reduction	Improved Healing	Ref.
Ex Vivo	Wound healing organ culture	Not infected	ALA	n.a.	YES	[68]
In vivo animal studies	Excisions	Not infected	ALA	n.a.	YES	[72]
			AlPcCl-PEG	n.a.	YES	[55]
		*E. coli*	pLC*e6*	YES	inconclusive	[98]
			Ce6-IL	YES	YES	[108]
			TCPP-PB@MOF	YES	YES	[118]
			TCPP-PCN224	YES	YES	[119]
			MOF-TCPP-Dopamine	YES	YES	[121]
			PGA-TMPyP nanofibrous mat	YES	YES	[122]
			AgNP/ZnPcCO_2_H fabric	YES	YES	[132]
		*P. aeruginosa*	pL-C*e6*	YES	YES	[99]
			pL–C*p6*	YES	NO	[110]
		*S. aureus*	Ce6-IL	YES	YES	[108]
			TCPP-PB@MOF	YES	YES	[118]
			TCPP-PCN224	YES	YES	[119]
			MOF-TCPP-dopamine	YES	YES	[121]
			PGA-TMPyP nanofibrous mat	YES	YES	[122]
		MRSA; *P. aeruginosa*	pL–C*p6*	n.d.	NO	[111]
			TMPyP	YES	inconclusive	[117]
		MRSA; *E. coli*; *P. aeruginosa*	TDAHPP	YES	YES	[116]
		MREC; MRAB	ZnPc-(Lys)_5_	YES	YES	[130]
		MRSA	RLP068/Cl-cellulose gel	YES	YES	[126]
	Abscesses	*S. aureus*	pLC*e6*	YES	inconclusive	[106]
		*E. coli*		YES	inconclusive	[106]
		*P. aeruginosa*		YES	inconclusive	[106]
		*A. baumannii*		YES	inconclusive	[100]
	Burns	Not infected	MAL	n.a.	YES	[89]
		MRSA	PEI-C*e6*	YES	YES	[101]
			RLP068/Cl	YES	YES	[127]
		MDR *S. aureus*	Sinoporphyrin derivative	YES	YES	[112]
		*S. aureus*	PTMPyP	YES	NO	[113]
	Ulcers	MRSA	ALA	YES	YES	[79]
	Skin wounds (not specified)	Not infected	Pheophorbide *a*	n.a.	YES	[97]
			Fotoditazin–Pluronic F127	n.a.	YES	[107]
		*P. aeruginosa*	ALA	YES	YES	[80,90]
		*S. aureus*	HTCC-C*e6*-Mg-EGCG NP	YES	YES	[109]
	Incisions	*Not infected*	m-THPC-collagen scaffolds	n.a.	YES	[114]
			CASP	n.a.	YES	[121]
			MPD-MA	n.a.	NO	[122]
In vivo human studies	Cutaneous Leishmaniasis lesions	*Leishmania major*; *Leishmania tropica*	ALA	n.d.	YES	[75] [76]
	Leg Ulcer	MRSA	ALA	n.d.	YES	[78]
		*P. aeruginosa*	ALA	n.d.	YES	[69]
			RLP068/Cl	YES	YES	[128]
		*S. aureus*; *P. aeruginosa* *E. coli*	TPP	YES	YES	[115]
		*S. aureus*	RLP068/Cl	YES	YES	[128]
	Chronic Venous Ulcers	*E. faecalis*	ALA	n.d.	YES	[81]
			MAL	n.d.	YES	[86]
		*S. aureus*	ALA	n.d.	YES	[81]
			MAL	n.d.	YES	[86]
	Actinic Keratosis	Not infected	MAL	n.a.	YES	[82]
	Excisional Wounds	Not infected	MAL	n.a.	inconclusive	[67]

n.a.—not applicable; n.d.—not determined.

## Data Availability

Not applicable.

## Abbreviations

AgSD	Silver sulfadiazine cream
ALA	5-Aminolevulinic acid
AlPcCl	Aluminium complex of phthalocyanine
BPD-MA	Benzoporphyrin mono-carboxylic acid
CASP	Chloroaluminum sulfonated phthalocyanine
C*e6*	Chlorin *e6*
C*p6*	Chlorin *p6*
CS	Chitosan
CVU	Chronic venous ulcers
DFUs	Diabetic foot ulcers
EDC	1-Ethyl-3-(3-dimethylaminopropyl)carbodiimide
EDTA	Ethylenediamine tetraacetic acid
EGCG	(–)-Epigallocatechin-3-gallate
FDA	Food and drug administration
GF	Growth factors
GTMAC	Glycidyltrimethylammonium chloride
HAL	Hexyl aminolevulinate
HpD	Hematoporphyrin derivative
ICCD	Intensified charge-coupled device
IL	Ionic liquid
LED	Light-emitting diode
LLLT	Low-level laser irradiation
MAL	Methyl aminolevulinate
MMP	Metalloproteinases
MOF	Metal-organic framework
MRAB	Multidrug-resistant Acinetobacter baumannii
MREC	Multidrug-resistant *Escherichia coli*
MDR	Multidrug-resistant
MRSA	Methicillin-resistant *Staphylococcus aureus*
*m*-THPC	5,10,15,20-Tetrakis(3-hydroxyphenyl)chlorin
NHS	*N*-hydroxysuccinimide
NMB	New Methylene Blue
NP	Nanoparticle
Pc	Phthalocyanines
PDI	Photodynamic inactivation
PF	Pluronic F127
pL-Ce6	Poly-L-lysine chlorin *e6*
pL-C*p6*	Poly-L-lysine chlorin *p6*
PB@MOF	Prussian Blue MOF
PEI	Polyethyleneimine
PP-IX	Protoporphyrin-IX
PTMPP	5-Phenyl-10,15,20-tris(1-methylpyridinium-4-yl)porphyrin trichloride
PVP	Polyvinylpyrrolidone
RB	Rose Bengal
ROS	Reactive oxygen species
SOD	Superoxide dismutase
TBO	Toluidine blue O
TCPP	5,10,15,20-Tetrakis(4-carboxyphenyl)porphyrin
TGF	Transforming growth factor
TLD141	[Ru(2,2′-bipyridine)2(2-(2′,2″:5″,2‴-terthiophene)-imidazo-[4,5-f][1,10]phenantroline]^2+^
TMPyP	5,10,15,20-Tetrakis(1-methylpyridinium-4-yl)porphyrin
TPP	5,10,15,20-Tetraphenylporphyrin
VC_12_IM	1-Vinyl-3-dodecylimidazolium bromide
VCM	Vancomycin
WHO	World Health Organization
WHOC	Wound healing organ culture
γ-PGA	Poly(γ-glutamic acid)
